# nNOS regulates ciliated cell polarity, ciliary beat frequency, and directional flow in mouse trachea

**DOI:** 10.26508/lsa.202000981

**Published:** 2021-03-02

**Authors:** Anatoly Mikhailik, Tatyana V Michurina, Krikor Dikranian, Stephen Hearn, Vladimir I Maxakov, Saul S Siller, Ken-Ichi Takemaru, Grigori Enikolopov, Natalia Peunova

**Affiliations:** 1 Center for Developmental Genetics, Stony Brook University, Stony Brook, NY, USA; 2 Department of Anesthesiology, Renaissance School of Medicine at Stony Brook University, Stony Brook, NY, USA; 3 Department of Neuroscience, Washington University, St. Louis, MO, USA; 4 Cold Spring Harbor Laboratory, Cold Spring Harbor, NY, USA; 5 Department of Pharmacological Sciences, Stony Brook University, Stony Brook, NY, USA

## Abstract

Clearance of the airway is dependent on directional mucus flow across the mucociliary epithelium, and deficient flow is implicated in a range of human disorders. Efficient flow relies on proper polarization of the multiciliated cells and sufficient ciliary beat frequency. We show that NO, produced by nNOS in the multiciliated cells of the mouse trachea, controls both the planar polarity and the ciliary beat frequency and is thereby necessary for the generation of the robust flow. The effect of nNOS on the polarity of ciliated cells relies on its interactions with the apical networks of actin and microtubules and involves RhoA activation. The action of nNOS on the beat frequency is mediated by guanylate cyclase; both NO donors and cGMP can augment fluid flow in the trachea and rescue the deficient flow in nNOS mutants. Our results link insufficient availability of NO in ciliated cells to defects in flow and ciliary activity and may thereby explain the low levels of exhaled NO in ciliopathies.

## Abstract (precis)

Directional flow of mucus across the mucociliary epithelium enables the clearance of the airway. This study shows that nNOS supports the flow by regulating the planar polarity and ciliary beat frequency of ciliated cells in the tracheal epithelium.

## Introduction

Airway clearance is crucial for the health of animals and humans and relies on the robust coordinated beating of cilia of the mucociliary epithelium that lines the airway. Efficient performance of cilia and the resultant propulsion of mucus serve as the airway’s first line of defense: foreign particles and microorganisms are trapped in the mucus, transported toward the larynx, and expelled from the airway. Defects in ciliary beating and mucus flow contribute to numerous disorders, including bronchiectasis, cystic fibrosis, asthma, chronic obstructive pulmonary disease, and primary ciliary dyskinesia (PCD) (Zariwala et al, 2007; Hildebrandt et al, 2011; Davis & Katsanis, 2012; Lobo et al, 2015; Werner et al, 2015; Bustamante-Marin & Ostrowski, 2016; Horani et al, 2016; Knowles et al, 2016; Boucher, 2019; Wheway & Mitchison, 2019).

Efficient mucus flow is gradually established during the development of the airway, with cilia beating with increased coordination and frequency as the mucociliary epithelium matures (Mitchell et al, 2007; Francis et al, 2009; Vladar et al, 2012). Coordinated beating and efficient flow require proper polarization and orientation of the multiciliated cells in the trachea as well as a proper spacing pattern and orientation of the cilia (Yoshimura et al, 2007; Hildebrandt et al, 2011; Werner et al, 2015). Mice are born with well polarized ciliated cells in the trachea, even though the refinement of cell polarity continues until up to 2 mo of age (Francis et al, 2009; Vladar et al, 2012). During embryonic development, the polarity of ciliated cells is guided genetically, primarily by core factors of the evolutionarily conserved planar cell polarity (PCP) pathway. PCP genes provide ciliated cells with global positional information, thus ensuring that the cells’ orientation in the trachea is aligned with the lung-to-larynx axis. Products of the PCP genes, including Dvl, Fz, Prickle, and Vangl, mark the proximal and distal sides of the cells and confer the initial positional bias (Wallingford, 2006, 2010; Wallingford & Mitchell, 2011; Vladar et al, 2012). Furthermore, regulators of actin and microtubule dynamics engage in cytoskeleton remodeling, which further drives cell polarization and determines the key features of planar polarity of ciliated cells: the spacing pattern of cilia and their orientation (rotational polarity) in the direction of the effective stroke.

As the airway matures, along with ciliated cell polarization, the ciliary beat frequency (CBF) increases, supported by the compressive and shear stress exerted by the airflow entering the airways with breathing (Button et al, 2007; Winters et al, 2007). Together, robust ciliary beat and proper arrangement and polarity of cilia contribute to the overall efficiency of the mucus flow across the mucociliary epithelium (Francis et al, 2009). No common factors that would regulate both the beat frequency of cilia and their distribution and orientation in the tracheal ciliated cells have been reported so far.

Nitric oxide (NO) is a plausible candidate that may integrate different modalities to modulate the activity of cilia in the ciliated epithelium. Neuronal isoform of NO synthase (nNOS/NOS1) has been detected in tracheal ciliated cells, and chemical NO donors added to tracheal ciliated cell preparations increase the CBF (Jain et al, 1993; Sisson et al, 2009; Jiao et al, 2011; Jackson et al, 2015). Furthermore, an nNOS ortholog regulates cell polarization during axis elongation in *Xenopus* embryos, a process that relies on the PCP pathway (Peunova et al, 2007). In addition, in the cardiovascular system, NO is produced by the endothelial isoform (eNOS/NOS3) in response to shear stress of the blood flow and rapidly remodels the cytoskeleton in smooth muscle cells, thus promoting vasorelaxation (Heiss et al, 2015; Shu et al, 2015); notably, nNOS isoform can similarly respond to shear stress by producing NO to support basal blood flow (Sato et al, 2008; Melikian et al, 2009; Hyndman et al, 2015; Zhang, 2016).

Remarkably, low levels of exhaled NO strongly correlate with PCD, a human disorder associated with cilia dysfunction and manifested in affected patients as a constellation of syndromes, such as chronic sinusitis, bronchiectasis, male and female infertility, reversal of left-right organ asymmetry, and hydrocephalus (Walker et al 2012, 2013; Lobo et al, 2015; Werner et al, 2015; Horani et al, 2016; Knowles et al, 2016). Notably, the link between insufficient NO production and poor cilia function is observed regardless of the specific genetic causes of the disorder. Even though the association between PCD and decreased production of NO is firmly established and is routinely used as a diagnostic tool for triaging PCD patients, the causal relationship between NO and PCD or the cellular mechanisms underlying this association are unknown.

Here, we investigate the roles of NO and nNOS in the functioning of cilia in the mouse tracheal mucociliary epithelium and show that nNOS has a versatile role in ciliated cells. First, it interacts with the core factors of PCP to translate the global planar polarity of the tracheal tissue into the polarity of individual ciliated cells and provides the cells with positional information in the tissue. In addition, NO/nNOS is important for the polarization of both the actin and microtubule apical networks, thus enabling correct spacing and orientation of the basal bodies and the cilia. Finally, we show that NO/nNOS, through activation of soluble guanylate cyclase (sGC), is necessary for supporting the CBF. Together, our results indicate a causal link between the cilia and flow dysfunction and the insufficient availability of NO.

## Results

### nNOS in the mouse trachea

**Figure 1.**
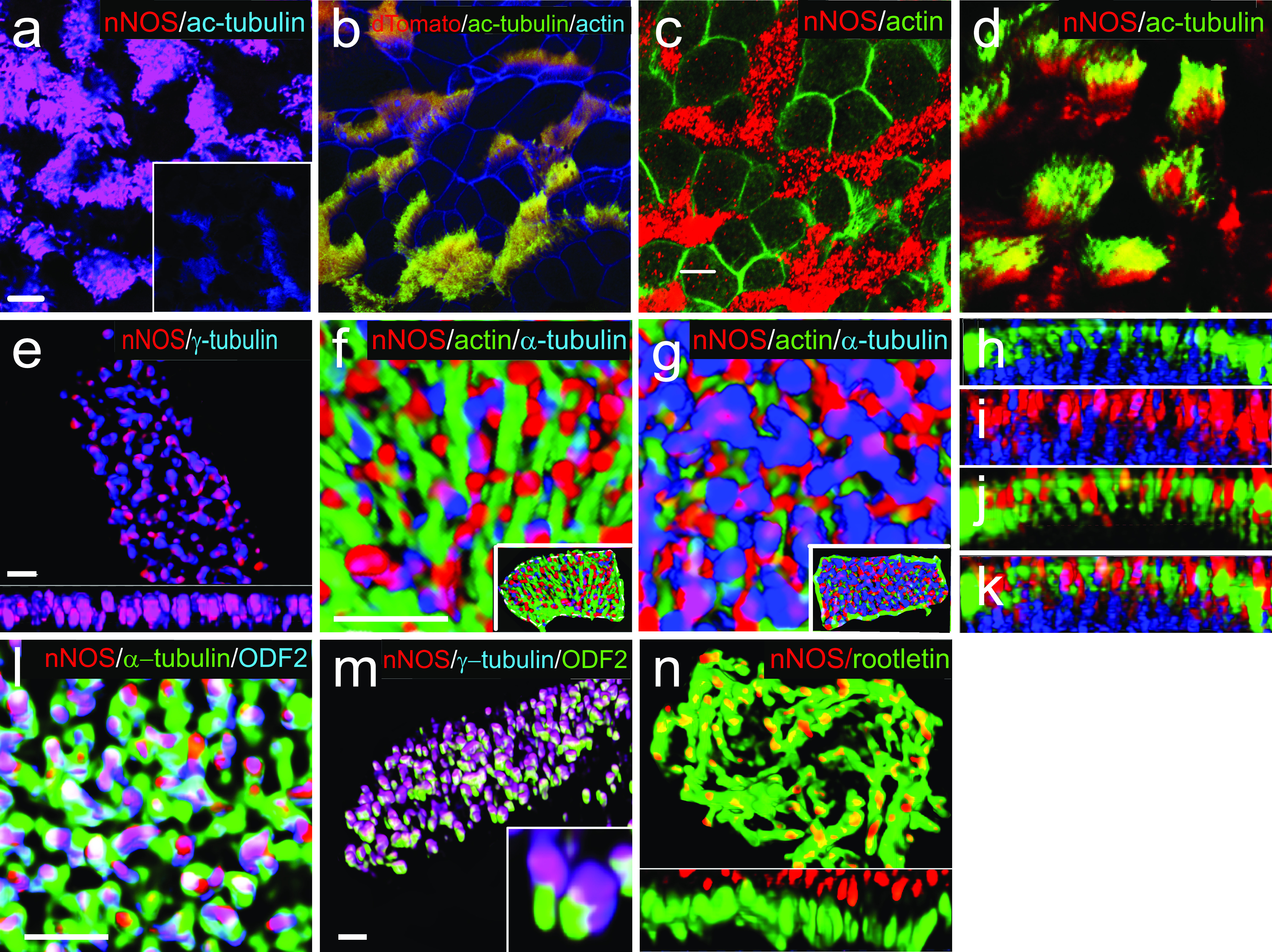
Localization of nNOS in the ciliated cells of trachea. **(A)** Immunostaining for nNOS shows its expression in the ciliated cells of the mouse trachea and absence in the nNOS-null mutant trachea (inset): nNOS: red; acetylated α-tubulin: blue (here and below the fluorophores for multiple labels are indicated on the figures by the corresponding colors). Scale bars are 5 μm in (A, B, C, D). **(B)** Detection of nNOS expression in the tracheal ciliated cells of nNOS-CreER/Ai9 mice. dTomato expression, driven by nNOS promoter, is observed in the ciliated cells, with the sample counterstained with phalloidin and acetylated α-tubulin. **(C)** nNOS is detected in the ciliary axonemes in the trachea, counterstained with phalloidin. **(D)** nNOS is detected in the apical cortex of the ciliated cell of the trachea and in the ciliary axonemes, stained with antibodies to nNOS and acetylated α-tubulin. **(E)** In cultured mouse tracheal ciliated cells nNOS is associated with basal bodies along their entire height. Z section from SIM in the inset. Scale bar is 2 μm. **(F, G, H, I, J, K)** In the ciliated cells of the mouse trachea, nNOS is associated with the cortical apical actin grid and cortical microtubules. Scale bar is 2 μm. **(F)** The image is focused on the apical-most area of the ciliated cells where nNOS is associated with the cortical actin grid; inset shows the entire cell (top view). **(G)** The same cell region as in (F), but flipped over to show the interaction of nNOS with the cortical network of microtubules located below the actin grid; inset shows the entire cell as in (F). **(H, I, J, K)** Z sections across the apical area of the ciliated cell of the tracheal explant, revealing the positions of the apical cortical actin grid and the microtubules (H); nNOS association with the cortical microtubules (I); nNOS association with the cortical actin grid (J); and nNOS association with both actin and microtubule components of the cortical cytoskeleton (K). **(L)** In the most basal aspects of the basal bodies nNOS is associated with microtubules and the basal feet (cultured mouse ciliated cells). Scale bars are 1 μm for (L, M, N). **(M)** nNOS is associated with basal bodies, labeled for γ–tubulin, and with basal feet, labeled for ODF2 (cultured mouse ciliated cells); higher magnification in the inset. **(N)** nNOS is not detected in the cilia rootlets in cultured ciliated cells labeled for rootletin. Z section in the inset.

Prompted by the association of low levels of NO with PCD (Werner et al, 2015; Knowles et al, 2016) and by the role of the nNOS ortholog in cell polarization and its interaction with the PCP pathway during *Xenopus* embryogenesis (Peunova et al, 2007), we investigated the role of nNOS in mouse tracheal multiciliated cells. Among the three NOS isoforms tested, only the nNOS signal was present in the mucociliary epithelium of the trachea (Fig 1), whereas the eNOS and iNOS signals were associated with the tracheal vasculature (data not shown). We compared images of the tracheas of the wild-type and nNOS-null mutant mice (nNOS knockouts; nNOS KO) (Packer et al, 2003) after immunostaining with antibodies to nNOS; we detected the nNOS signal in the wild-type tracheal ciliated cells but not in cells of the mutant animals (Fig 1A). Additional evidence that nNOS is expressed in ciliated cells was obtained by genetically marking the nNOS-expressing cells: after crossing nNOS-CreER driver and Ai9 reporter mouse lines and inducing recombination with tamoxifen, we found that ciliated cells in the trachea in the nNOS-CreER/Ai9 mice (Taniguchi et al, 2011) expressed nNOS promoter-driven fluorescent protein (Fig 1B). Thus, immunocytochemical and genetic labeling approaches demonstrated that nNOS, but not the other NOS isoforms, is expressed in mouse tracheal multiciliated cells.

We detected nNOS in ciliary axonemes (Fig 1C) and in the apical zones of ciliated cells (Fig 1D), where the basal bodies interact with apical actin and microtubule cytoskeletons (Werner et al, 2011; Kunimoto et al, 2012; Vladar et al, 2012). We then applied structured illumination superresolution microscopy (SIM) to investigate the details of interactions between nNOS, the basal body apparatus, and the cytoskeleton of ciliated cells.

SIM revealed that the apical actin cortex of tracheal ciliated cells forms a ∼2-μm deep grid with openings for the ciliary axonemes, with the basal bodies sandwiched between the apical actin grid at their upper limit and the network of cortical microtubules at their lower limit (Fig 1E–K). The nNOS signal was associated with the basal bodies and distributed along their entire height (Fig 1E). Within the apical aspect, the nNOS signal extended to the actin grid (Fig 1F, H, and J), whereas in the basal aspect, it reached to the microtubule network (Fig 1G, I, and K). The nNOS signal reached the basal foot, an appendage of the basal body that anchors microtubules, and it partially overlapped with the signal of ODF2, an essential component of the basal foot (Fig 1L and M). The zone of nNOS distribution did not overlap with the distribution of rootletin, a marker of cilia rootlets (Fig 1N). Overall, the conspicuous distribution of nNOS with respect to the basal bodies and the components of the apical cytoskeleton, as well as its presence in the axonemes, prompted us to investigate the possible involvement of nNOS in the polarization of tracheal ciliated cells and ciliary function.

### Ciliated cells in nNOS knockout mouse trachea

**Figure 2.**
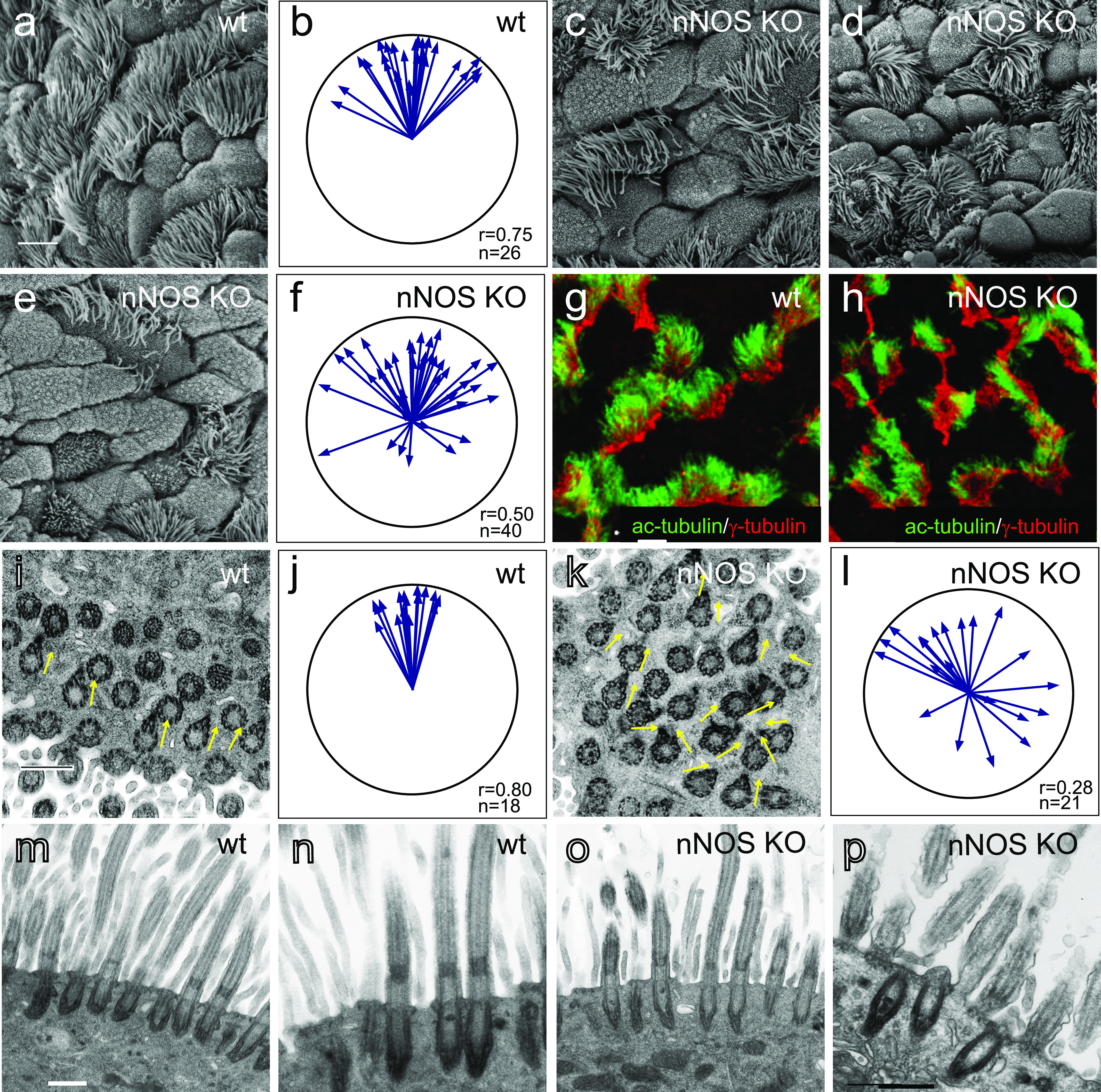
Distortion of the ciliated cell polarity in the trachea of nNOS-null (nNOS KO) mice. **(A)** Scanning electron microscopy (SEM) of the ciliated cells in the wild-type mouse trachea. Scale bar is 5 μm in (A, C, D, E, G, H). **(B)** Circular plot of cilia orientation in wild-type tracheal ciliated cells. Direction of the arrow represents the mean vector of the cilia orientation for a given cell, with the larynx position corresponding to the top, and the arrow length represents the value of the mean vector (longer arrows indicating higher coordination of cilia orientation for that cell); r describes rotational orientation of the cilia, n:number of evaluated cells. **(C, D, E)** SEM of the ciliated cells in nNOS KO mice. Note grossly misshapen cells with sparse cilia (C); cells with rosette-like arrangement of cilia (D); and cells with short stub-like cilia (E). **(F)** Circular plot of cilia orientation in nNOS mutant tracheal ciliated cells, visualized by SEM. **(G, H)** Immunocytochemical staining for basal bodies (γ-tubulin) and cilia (acetylated α-tubulin). **(G, H)** nNOS KO cells show defects in cilia orientation, chaotic spacing pattern, and short and sparse cilia (G) as compared with cilia organization in the wild type (H). **(I, J, K, L)** Transmission electron microscopy. **(I, K)** Cilia in the wild-type (I) and nNOS mutant (K) trachea, with arrows indicating basal feet orientation. **(I, K)** Scale bar is 0.5 μm in (I and K). **(J, L)** Circular plots, characterizing rotational polarity of basal bodies shows coordination in the cilia orientation in the wild type (J), as compared with the nNOS mutant (L). **(M, N, O, P)** Transmission electron microscopy of a cross sections of the trachea: there were no detectable defects in the docking of the basal bodies to the cell membranes (M, O); however, abnormal basal bodies associated with abnormal short axonemes and not generated axonemes were observed in nNOS KO cells (P) in comparison to the wild type (N). Scale bar is 0.5 μm in (M, N, O, P).

The spacing and orientation of the basal bodies are the crucial features of the polarity of ciliated cells in the trachea. To investigate the role of nNOS in ciliated cell polarity, we compared the tracheal mucociliary epithelium in wild-type and nNOS-deficient mice. We did not detect differences in the ratio of ciliated cells to other cell types in the epithelium, thus indicating that nNOS is not essential for the determination of cell fate in the developing mucociliary epithelium. However, the morphology of ciliated cells, as analyzed by scanning electron microscopy, was notably different in the nNOS KO than the wild-type animals (Fig 2A–F). In the trachea of wild-type mice, the cilia were of consistent length, and their orientation was coordinated with adjacent cells, with a bias toward the direction of the presumptive mucus flow, in general alignment with the long (AP; lung-to-larynx) axis of the trachea (Fig 2A and B). In the nNOS KO mouse trachea, a wide range of defects was observed (Fig 2C–F). Many ciliated cells were grossly misshapen, with the average apical area almost twice that in the wild type (110 ± 20 versus 62 ± 10 μm^2^) (Fig 2C). In addition, numerous nNOS KO ciliated cells had cilia that were sparser and shorter than those in the wild type (3.8 ± 0.5 μm in nNOS KO versus 5.4 ± 0.6 μm in the wild type) and frequently bore very short stub-like cilia (the length of axonemes was too short to measure accurately) (Fig 2C–E).

The orientation of cilia in the nNOS KO trachea was poorly coordinated, although the degree of this defect varied: in the mild phenotype, the orientation of the cilia within single cells showed coordination; however, poor alignment with the adjacent cells was observed. In the severe phenotype, the ciliary orientation was poorly coordinated even within single cells, and the cilia were often organized in a rosette-like pattern (Fig 2D). Besides the deformed cells, there were also groups of cells present with apparently normal cilia polarity. The penetrance of the mutants phenotype also differed between individual animals. In most cases, most of the ciliated cells in a tracheal preparation would carry distinct and severe defects in polarity, with mildly or minimally affected cells observed in the same preparation. However, a quarter of all animals showed particularly severe defects of the tracheal ciliated cells, with essentially no normal cells in sight; in a small fraction of animals (5–7%) manifestation of the mutant phenotype was very weak.

The results of scanning electron microscopy were supported by confocal microscopy after immunocytochemical staining for cilia and basal body markers, which similarly showed that in the nNOS KO trachea, the orientation of the cilia and their spacing pattern were disorganized, and the cilia were shorter, sparser, and not aligned with the direction of airway clearance (Fig 2G and H).

The orientation of the cilia in the direction of flow is established by rotation of the basal body, with the basal foot being correctly oriented. We applied transmission electron microscopy (TEM) to determine the orientations of the basal feet in horizontal sections of the trachea, and found that in the nNOS KO samples, the orientation of the basal bodies within single ciliated cells and between adjacent cells was less coordinated than that in the wild type (Fig 2I–L). The fewer cilia observed at the surfaces of ciliated cells of the mutants may potentially have been due to a failure of docking of the basal bodies to the apical membrane (e.g., basal bodies stalled deeper in the cytoplasm). TEM of cross-sections of the mutants’ tracheas did not indicate basal bodies deep in the cytoplasm (Fig 2M and O). However, we detected ciliated cells with short abnormal cilia and with the basal bodies located in the immediate vicinity of the apical membrane but not associated with normal long axonemes (Fig 2N and P), indicating that nNOS may be involved in the growth of cilia.

**Figure 3.**
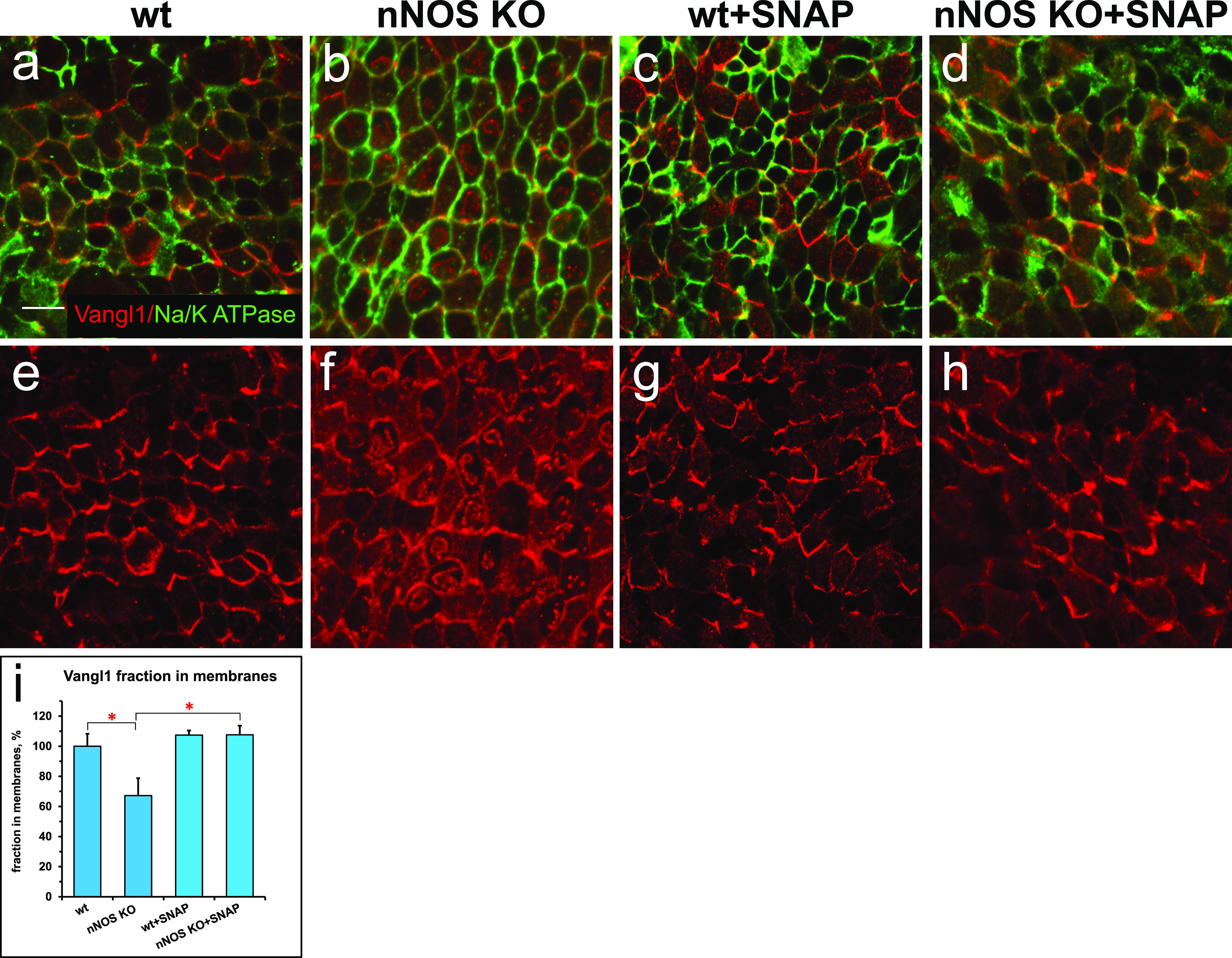
Distortion of the planar cell polarity in the trachea of nNOS KO mice. **(A, E)** Planar polarity of the ciliated cells in the wild-type tracheal cells, characterized by the asymmetric distribution of Vangl1, which is concentrated at the membranes at the distal aspect of ciliated cells; membrane is marked by antibody to Na^+^/K^+^ ATPase. **(B, F, I)** The membrane enrichment of Vangl1 is distorted in the ciliated cells of the tracheal explant of nNOS KO, as manifested by diminished association of the protein with the membranes and its excessive accumulation in the cytoplasm. **(C, D, G, H, I)** SNAP treatment for 30 min improves the membrane association of the Vangl1 protein in the mutant, but not the wild-type tracheal explants. Bar in (I) shows mean ± s.e.m. *P < 0.05, **P < 0.01. Scale bar is 5 μm.

Given the importance of the PCP pathway for multiciliated cell polarization (Wallingford, 2006, 2010; Wallingford & Mitchell, 2011; Vladar et al, 2012), we examined the changes evoked by NO on the distribution of the core PCP pathway factor Vangl1 (Kunimoto et al, 2012; Vladar et al, 2012). We compared the patterns of Vangl1 in wild-type and nNOS KO tracheas. In the wild-type animals, Vangl1 protein was found in the membranes and was localized asymmetrically, as a crescent at the distal sides of ciliated cells (facing the lungs); this stereotypic polarized pattern was common among neighboring cells (Fig 3A, E, and I). In the nNOS KO trachea, a smaller fraction of Vangl1 protein was localized at the cell membranes, whereas a substantial fraction of Vangl1 accumulated as aggregates throughout the cytoplasm (Fig 3B, F, and I).

We next examined whether the treatment of trachea explants from wild-type and nNOS KO mice with the chemical NO donor S-nitroso-N-acetyl-penicillamine (SNAP) might evoke changes in the Vangl1 distribution in ciliated cells. Exposure of the wild-type trachea to 100 μM SNAP did not affect the polarity of the Vangl1 distribution or its localization to membranes (Fig 3C, G, and I). In contrast, SNAP treatment of nNOS KO trachea explants significantly rescued Vangl1 protein localization to the membranes, decreasing the fraction of cytoplasm-associated protein (Fig 3D, H, and I). These results indicate that establishment of the ciliated cell polarity mediated by the core factors of the PCP pathway is sensitive to NO signaling.

### Impaired fluid flow and ciliary beating in the nNOS KO trachea

Having found that nNOS is important for the polarity of ciliated cells, we examined the role of nNOS in the ability of cilia to generate effective directional flow. To investigate the flow dynamics, we used a live imaging assay to follow and quantify the movement of fluorescent beads along the preparations of dissected tracheas, which were longitudinally cut, flattened, and placed in a viewing chamber under a microscope. We analyzed the following parameters of the beads’ movement:

flow velocity: the net displacement of the beads, that is, the distance between the first and the last point of the path traveled by the bead per second;
overall trajectory traveled by a bead per second, and its deviation from the net displacement;
direction of the bead’s movement (vector of the net displacement); and
coordination of vectors of the net displacement of individual beads as the deviation from the median of the vectors of all analyzed beads.

**Figure 4.**
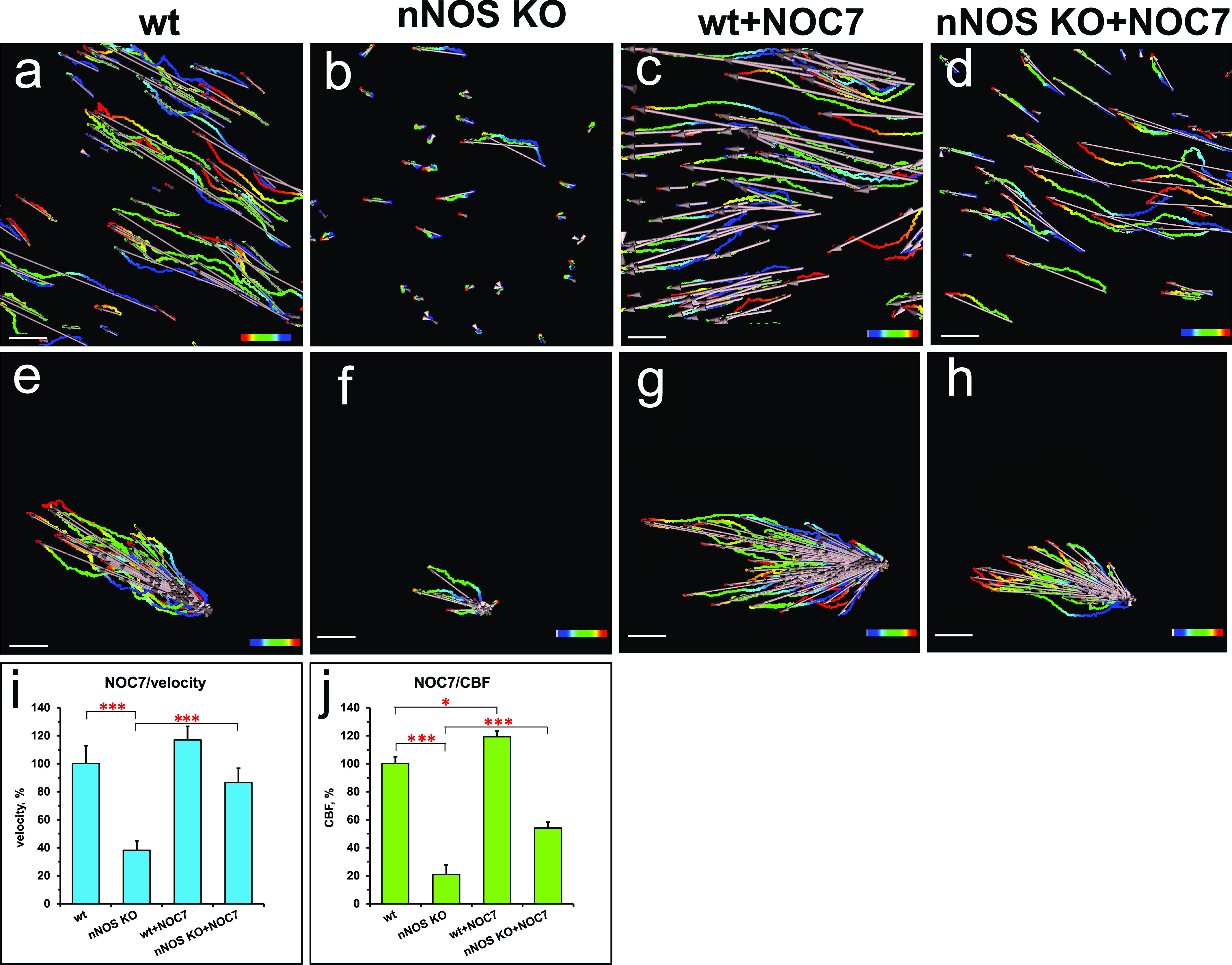
Nitric oxide is essential for polarized flow. **(A, B, C, D)** Spot tracking of beads movement in the wild-type (A) and nNOS KO (B) trachea. Defective flow in the nNOS mutant (B) can be improved by the addition of NO donor NOC7 (D). NOC 7 also increases the flow velocity in the wild type (C). Scale bar is 50 μm. **(E, F, G, H)** The trajectory path (colored), net displacement, and direction of the beads in the flow (white arrow) probed in the wild-type (E) and nNOS KO (F) trachea specimens before (E, F) and after (G, H) addition of NOC7. **(I, J)** Effect of NOC7 on ciliary beat frequency and flow velocity is summarized in charts (I, J). Scale bars are 50 μm. Corresponding Videos 1–3 are presented in Supplemental Material.

In the wild-type trachea, the fluorescent beads moved rapidly (46.8 ± 4.4 μm/s; mean ± sem), with an optimal trajectory (i.e., the overall path traveled by a bead close to its net displacement) and well-coordinated directionality (with the bulk of the beads’ movement vectors close to the median direction) (Fig 4A, E, and I).

A lack of nNOS impaired all analyzed flow parameters. In particular, in the trachea in nNOS mutant mice, the flow velocity was diminished twofold to 22.6 ± 3.6 μm/s, and the beads moved in a poorly coordinated manner (Fig 4B, F, and I).

CBF is a critical contributor to flow efficiency. Because chemical NO donors have been reported to stimulate the ciliary beat (Jain et al, 1993; Sisson et al, 2009; Jiao et al, 2011), we examined the contribution of nNOS to the CBF. In these experiments, cilia were visualized by live staining with fluorescein-conjugated wheat germ agglutinin. This method allowed us to follow changes in the orientation of cilia beating and beads movement in a live tissue in parallel with measuring the CBF values.

In the trachea in wild-type animals, most cilia beat in unison with a consistent frequency (9.8 ± 0.84 Hz) and in a coordinated manner, generating an efficient directional flow (Fig 4J and Video 1). In the nNOS KO trachea, the CBF was significantly lower (2.1 ± 0.75 Hz) (Fig 4J), and the beating pattern showed profound uncoordination between neighboring cells and often within single ciliated cells. Moreover, we observed numerous cells with very slowly moving and even immotile cilia (5–20% in trachea samples from different mutant animals) (Video 2); however, we also observed rare areas with preserved coordination of the ciliary movement between ciliated cells in the nNOS mutants.

Video 1 Nitric oxide donor NOC7 augments ciliary beat frequency and flow velocity in the trachea explants of wild-type animals. Download video

Video 2 Nitric oxide donor NOC7 increases ciliary beat frequency and flow velocity in the trachea explants of nNOS KO animals. Download video

**Figure 5.**
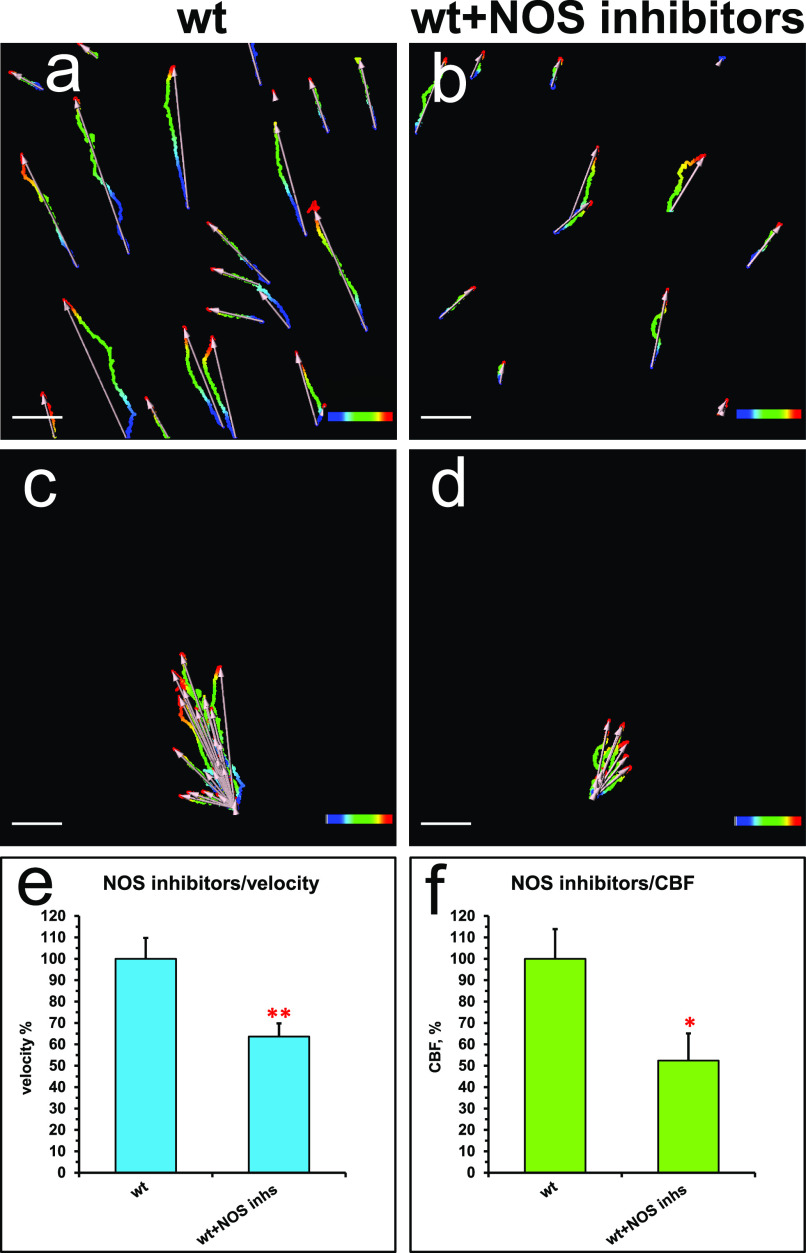
Inhibitors of NOS decrease flow velocity and ciliary beat frequency (CBF). **(A, B, C, D, E, F)** Spot tracking of beads movement and CBF measurement in the wild-type trachea after exposure to NOS inhibitors. **(A, B, C, D, E, F)** Incubation of the preparations for 30 min with a mix of 1 mM L-NAME, 100 μM ETU and 100 μM S-methyl-L-thiocitrulline hydrochloride (SMTC) resulted in a decrease in all fluid flow parameters (B, D) in comparison with non-treated control (A, C), demonstrating inhibitory effects both on the flow velocity and CBF (charts in E, F).

Similarly to the effects of the nNOS deletion, the addition of NOS inhibitors to the wild-type tracheal preparations decreased the flow velocity and CBF, to 63% and 52%, respectively, of the wild-type values (Fig 5A–F).

### NO donors restore directional flow and ciliary beating

We next asked whether the lack of functional nNOS enzyme in nNOS KO trachea might be compensated by exogenous NO released by chemical NO donors. We found that after the addition of 10 μM 3-(2-hydroxy-1-methyl-2-nitrosohydrazino)-N-methyl-1-propanamine (NOC7) to the tracheal preparations of nNOS KO mice, the velocity and CBF significantly increased, 1.6-fold and 2.6-fold, respectively (Fig 4D and H–J). In the wild-type trachea explants, NOC7 also increased the flow velocity and CBF, to 128% and 127%, respectively (Fig 4C, G, I, and J and Videos 1 and 2). Notably, in the nNOS KO preparations exposed to NOC7, we no longer observed areas with immotile cilia. However, we did not observe an obvious rescue of cilia beating coordination throughout the tissue, commonly detecting areas where cilia were moving with poor coordination (albeit with higher CBF) after NOC7 treatment.

**Figure S1.**
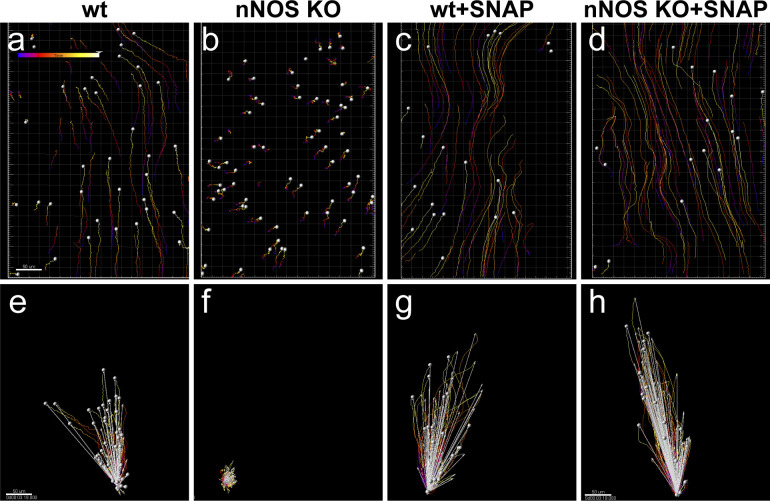
Nitric oxide donor SNAP replicates the effect of the flow rescue by NOC7; nitric oxide is essential for polarized flow. **(A, B, C, D, E, F, G, H)** Spot tracking of beads movement and analysis of the beads trajectory movements, net displacement and direction of the flow in the wild-type (A, E) and nNOS KO (B, F) trachea demonstrate defective flow in the nNOS mutant. **(C, D, G, H)** SNAP rescues the flow velocity and direction in tracheal explants from nNOS KO mutants (D, H) and facilitates the flow in the wild-type explants (C, G). **(E, F, G, H)** show the distance (red), net displacement (white) and direction of the beads’ movement. Scale bar is 50 μm. Corresponding movie is presented in Supplemental Material as Video 3.

We also found that a chemically distinct NO donor, SNAP, which has a longer half-life than NOC7 (5 h versus 10 min at room temperature), elicited similar changes in the preparations of wild-type and mutant tracheas, thereby effectively rescuing the flow velocity and CBF in the nNOS KO (Fig S1A–H and Video 3).

Video 3 Nitric oxide donor SNAP increases flow velocity in the wild-type trachea and rescues deficient flow in the nNOS KO trachea. Spot tracking of the beads movement driven by the flow generated by cilia beating, in the explants of the wild-type trachea (A); wild-type trachea treated with NO donor SNAP (B); trachea of nNOS KO (C); and trachea of nNOS KO treated with NO donor SNAP (D). Download video

Together, these results indicated that nNOS is critical for the efficiency of the CBF and for the velocity and directionality of the generated fluid flow; that exogenous NO donors can increase velocity and CBF in wild-type tracheal explants; and that NO donors are able to mitigate the defects in flow and CBF induced by the loss of nNOS.

### cGMP mediates the action of nNOS in ciliated cells

sGC is a key effector of NO in a range of biological settings: binding of NO to the heme-containing catalytic center activates the enzyme and leads to a rapid increase in the cGMP level, which in turn activates cGMP-dependent kinase, with subsequent phosphorylation of target proteins. Therefore, to determine whether the observed effects of NO might be mediated by the cGMP signaling pathway, we examined the effects of an sGC inhibitor and a cGMP analog on both the wild-type and mutant tracheas.

**Figure 6.**
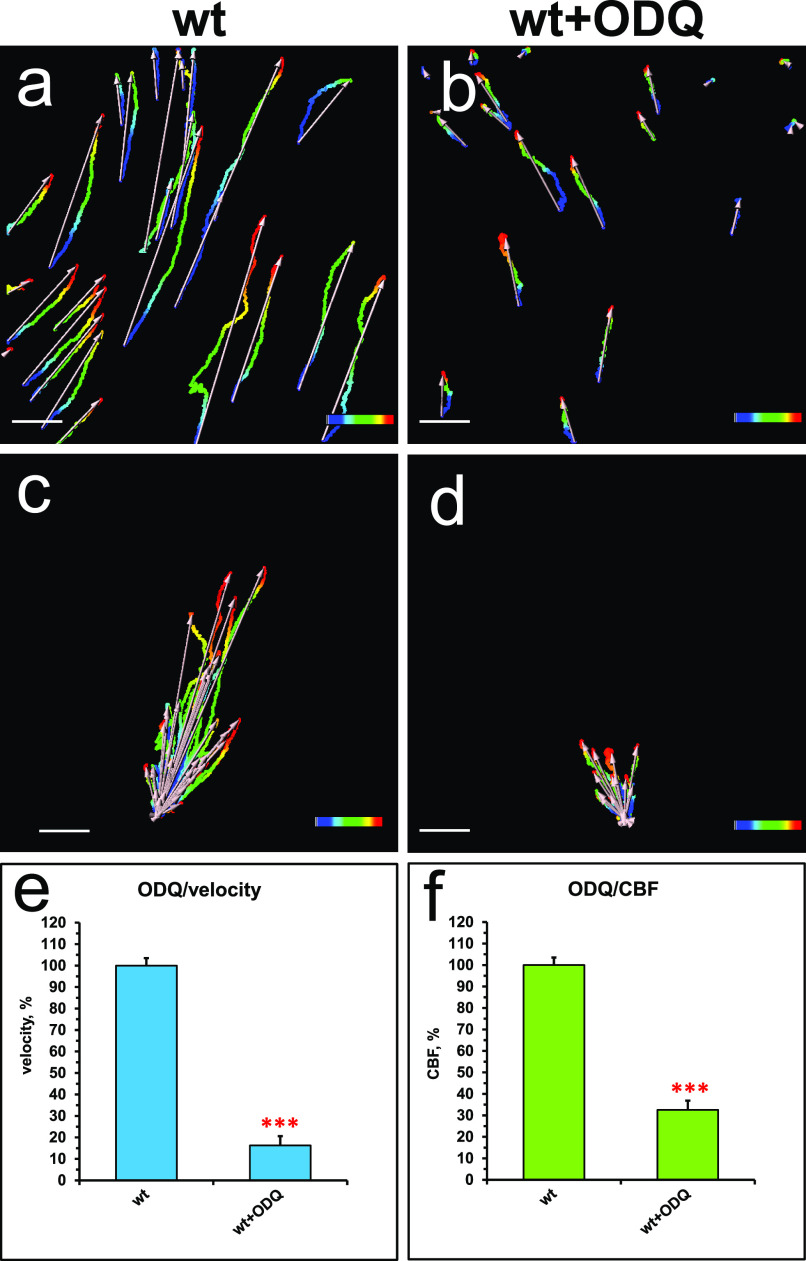
Soluble guanylate cyclase (sGC) is a necessary factor of the nitric oxide signaling pathway in controlling the fluid flow dynamics. **(A, B, C, D, E, F)** Spot tracking of beads movement and ciliary beat frequency measurement in the wild-type trachea after exposure to sGC inhibitor. Treatment of the specimens of wild-type trachea explants with an inhibitor of sGC, [1H-[1,2,4]oxadiazolo-[4,3-a]quinoxalin-1-one], ODQ for 30 min decreases the flow velocity and the ciliary beat frequency (B and D, E, F) in comparison with control (A and C, E, F). Corresponding Video 4 is presented in Supplemental Material.

Exposure of the wild-type trachea explants to the sGC inhibitor 1H-[1,2,4]-oxadiazolo-[4,3-alpha]-quinoxalin-1-one (ODQ) decreased the flow velocity sixfold and the CBF threefold (Fig 6A–F), thus resembling the results observed with the nNOS deletion and NOS inhibitors (Figs 4 and 5 and Video 4).

Video 4 ODQ, inhibitor of soluble guanylate cyclase, decreases ciliary beat frequency, slows down flow, and decreases cilia beat coordination within and between the ciliated cells of the trachea explants. Download video

**Figure 7.**
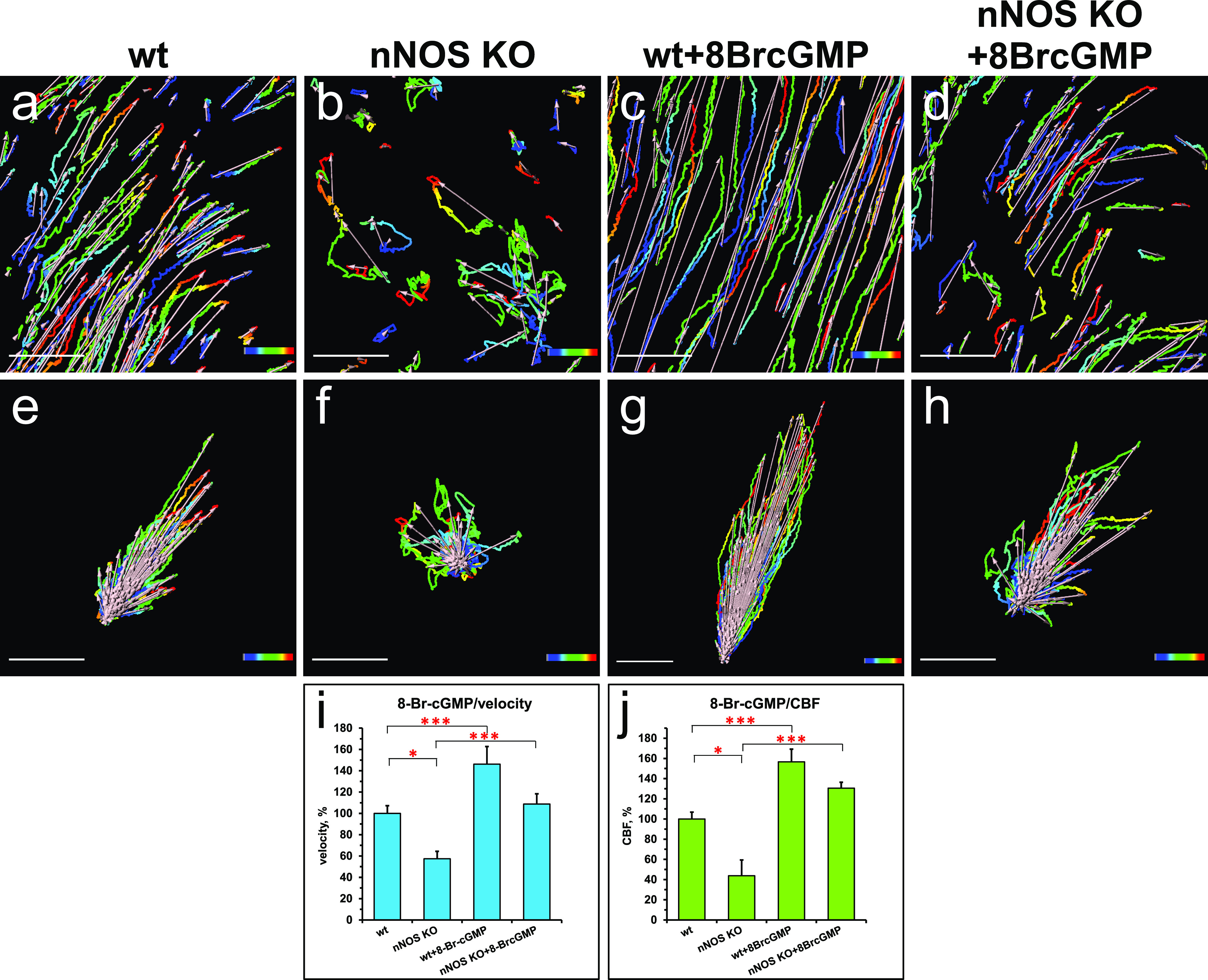
Nitric oxide positively controls ciliary beat frequency (CBF) and fluid flow through soluble guanylate cyclase-cGMP pathway. **(A, B, C, D, E, F, G, H, I, J)** Treatment of the tracheal explants with cGMP analog 8BrcGMP for 30 min replicated the effect of nitric oxide donors, rescuing the CBF and flow defects in the nNOS KO trachea. **(A, B, D, E, I, J)** Fluid flow and CBF in nNOS KO trachea, showing lower velocity, lower CBF, and poor directionality in comparison with the wild type (A, B, D, E, I, J) are significantly improved by the treatment with 8-Br-cGMP. 8-Br-cGMP also augments CBF and flow velocity in the wild-type trachea explants. Corresponding Videos 5 and 6 are presented in Supplemental Material.

We then asked whether a cell-permeant cGMP analog, 8-bromo-guanosine 3′,5′-cyclic monophosphate (8-Br-cGMP), might replicate the effects of the NO donors in the wild-type and mutant trachea. Addition of 8-Br-cGMP to the wild-type trachea explants increased the flow velocity 1.46-fold and the CBF—1.57-fold (Fig 7A, C, E, G, I, and J and Video 5). When added to the mutant trachea explants, 8-Br-cGMP increased the flow velocity 1.9-fold and the CBF threefold, thus bringing them close to the basal levels observed in the wild-type tracheas (Fig 7B, D, F, and H–J and Video 6).

Video 5 cGMP analog 8-Br-cGMP augments ciliary beat frequency and flow velocity in the trachea explants of wild-type animals. Download video

Video 6 cGMP analog 8-Br-cGMP increases ciliary beat frequency and flow velocity in the trachea explants of nNOS KO animals. Note that 8-Br-cGMP does not change the orientation of the beating of the cilia in cells with uncoordinated ciliary beat. Download video

Together, these results showed a strong response of both wild-type and nNOS-deficient tracheal ciliated cells to the sGC inhibitor and the cGMP analog, thus pointing to the nNOS-NO-sGC-cGMP pathway as a critical effector of nNOS signaling in ciliated cells.

At the same time, these experiments leave open the question of whether the stimulating effect of the cGMP analog on the flow dynamics might be associated with the increase in CBF, the improved coordination of the ciliary beating, or both. To answer these questions, we modified the experimental setup to follow the behavior of the same group of ciliated cells as they responded to a particular treatment. We placed the tracheal preparations in a perfusion camera with the medium pumped through the camera at a low speed of 5 μm/s — conditions allowing the tissue to stay alive for hours, with CBF maintained at consistent physiological levels, and without interfering with the velocity of cilia-generated flow (Winters et al, 2007). In addition, a T-valve switch, set next to the chamber, allowed for rapid replacement of the medium with one containing the 8-Br-cGMP and thus enabled continued recording the responses of the same group of ciliated cells to the treatment.

**Figure 8.**
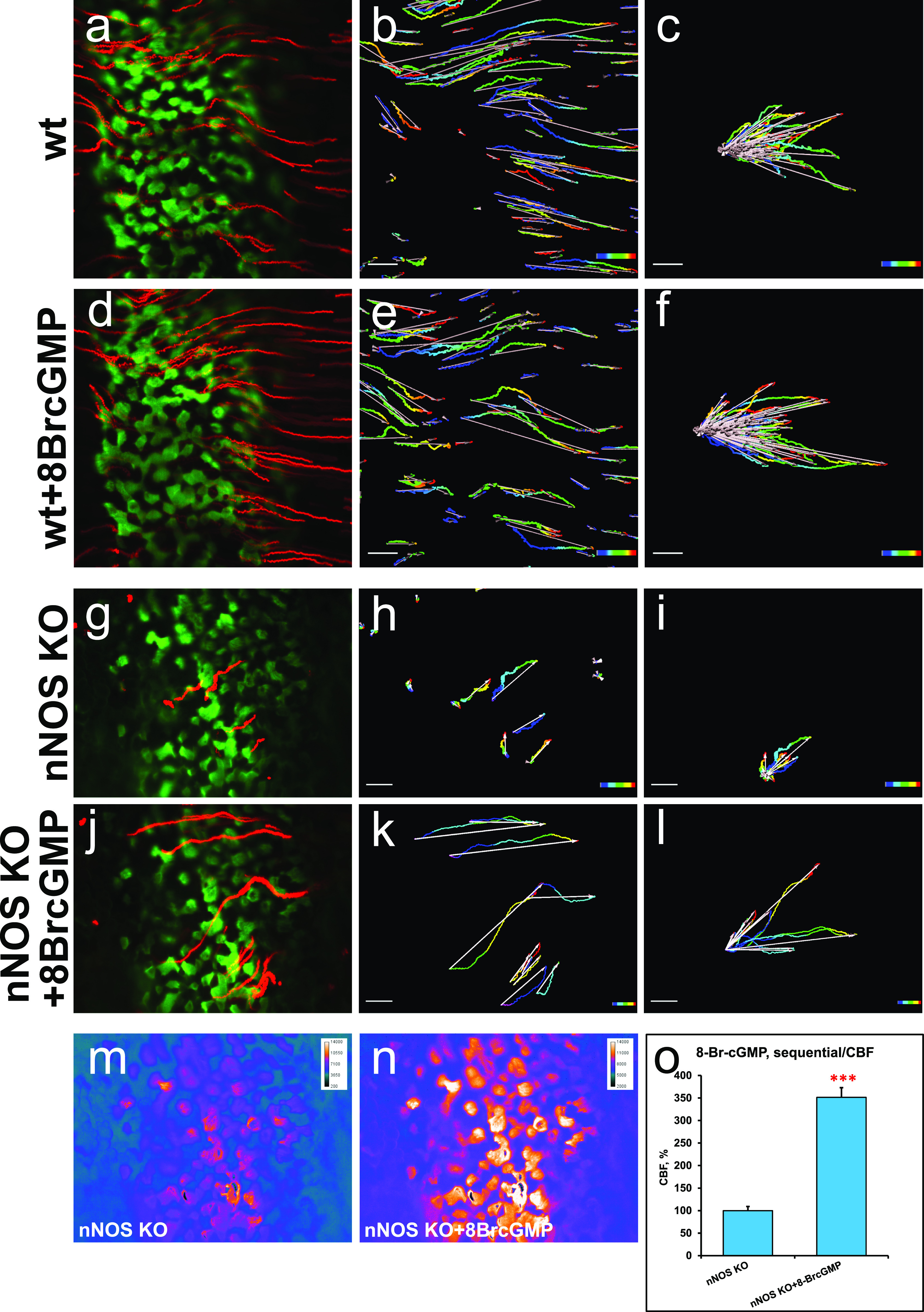
Increase of the ciliary beat frequency is the primary effect of 8-Br-cGMP treatment in rescuing fluid flow in nNOS KO tracheal explants. **(A, B, C, D, E, F, G, H, I, J, K, L, M, N, O)** Focusing on the same area of the tracheal ciliated epithelium in the wild-type and nNOS KO trachea and recording changes in flow velocity (by tracking beads’ movement) and cilia movements in individual ciliated cells before and after addition of 8-Br-cGMP. **(A, B, C, D, E, F, G, H, I, J, K, L)** Such sequential recording shows an increase of flow velocity induced by 8-Br-cGMP (revealed as longer tracks of the beads’ movements) in the wild-type (A, B, C, D, E, F) and nNOS KO (G, H, I, J, K, L) trachea. **(M, N, O)** It also shows an increase of ciliary beat frequency, presented as heat maps, in most individual cells across the area 5–10 s after addition of 8-Br-cGMP. Corresponding Video 7 is presented in Supplemental Material.

We first recorded ciliary beating while explants were perfused with regular medium, then switched to perfusion with medium containing 8-Br-cGMP, focusing on the same area. We found that, as in the basic setup, addition of 8-Br-cGMP to the wild-type tracheal preparations significantly increased the flow velocity and CBF, ∼1.5-fold for both parameters (Fig 8A–F).

We then used this sequential recording setup to analyze the nNOS KO tracheal preparations, focusing on the representative areas that showed various types of defects in ciliary function, as described earlier: (i) slower overall ciliary beating; (ii) cells with immotile cilia; (iii) cells lacking coordination with the neighboring cells in their beating direction; and (iv) cells with cilia still beating in apparent coordination with their close neighbors. As with the basic setup, addition of the cGMP analog increased the flow velocity and CBF in nNOS-deficient cells (Fig 8G–L and Video 7).

**Figure 9.**
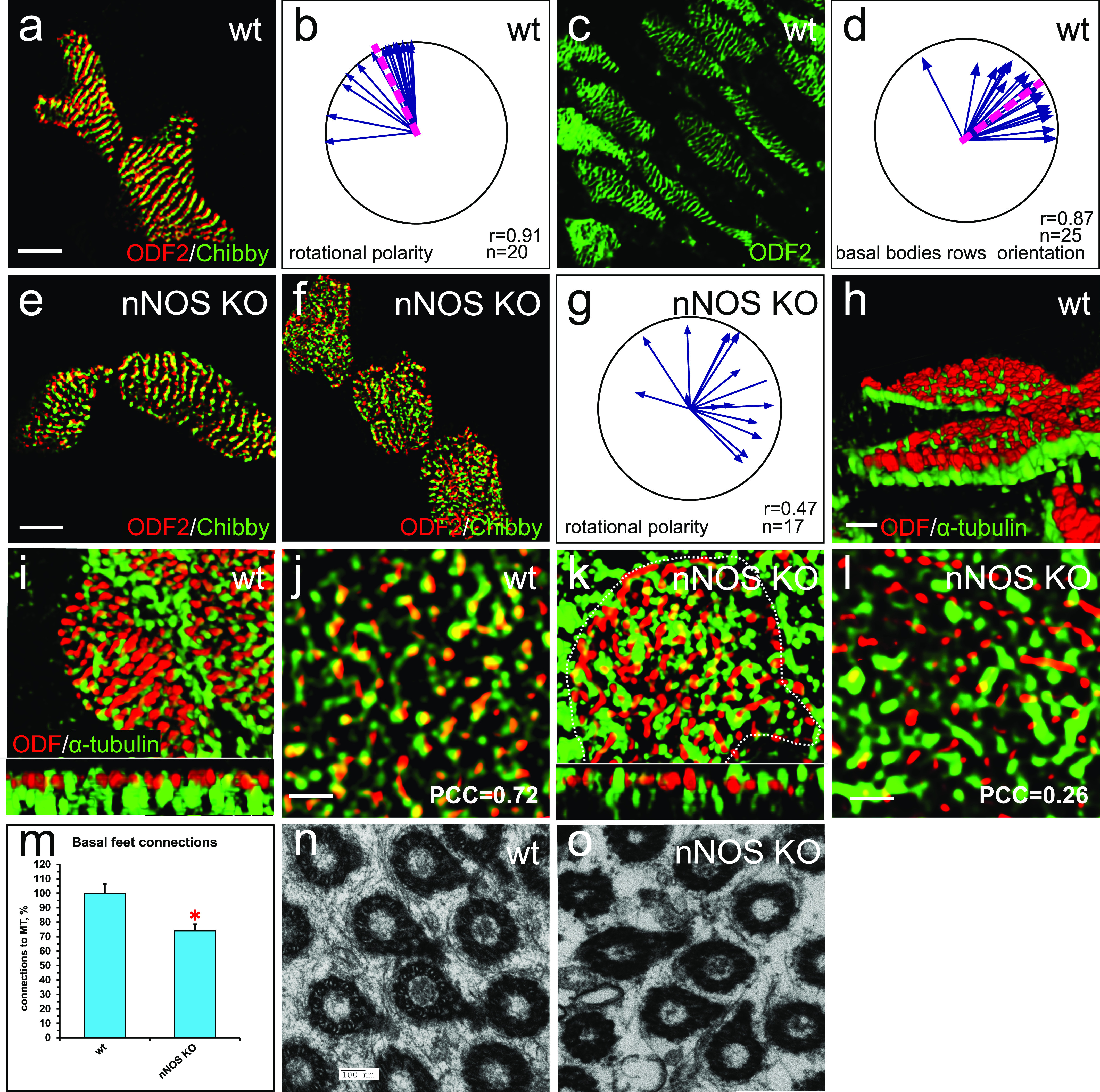
nNOS is essential for cilia spacing pattern and rotational polarity. **(A, B)** Cby1/ODF2 pair demonstrates high degree of coordination of rotational polarity within single ciliated cells and between neighboring cells in the trachea and alignment with the direction of the flow. Scale bar is 5 μm in (A, B, C, D, E, F). **(C, D)** ODF2 staining reveals the spacing pattern of basal bodies as parallel rows, oriented perpendicularly to the direction of the flow. **(E)** nNOS KO mutant cells with mild phenotype have preserved rotational polarity and spacing pattern of the basal bodies within single cells, which is poorly coordinated with the neighboring cells. **(F, G)** nNOS KO mutant ciliated cells with more severe phenotype lack rotational polarity both within the cell and between neighboring ciliated cells, and the spacing pattern of the basal bodies is scrambled. **(H, I, J)** Microtubules are anchored to the most basal aspects of the basal feet (labeled by ODF2) in the ciliated cells in the trachea (H, scale bar is 1 μm). Microtubules connect all basal bodies into a regular joint network in the ciliated cells of the trachea in wild type (I); (J) – higher magnification. **(K, L)** In the nNOS KO cells basal feet are aberrantly connected with microtubules, with some basal feet having excessive connections with the microtubules and others left out of the connections. **(H, I, J, K, L)** Scale bars are 1 μm in (H, I, K) and in (J, L). Pearson correlation coefficient for the overlap between fluorescent signals of ODF2 (Alexa-568) and microtubules (Alexa-488) is indicated. **(M)** Fraction of the basal feet connected to the microtubules in wild-type and nNOS KO ciliated cells (20 and 25 cells of each genotype analyzed, correspondingly). **(N, O)** Transmission electron microscopy shows a sparse network of microtubules connecting basal feet in nNOS KO, leaving some basal feet out of the network, as compared with wild type.

Video 7 cGMP analog 8-Br-cGMP increases ciliary beat frequency and flow velocity in the trachea explants of nNOS KO animals in sequential recording experiments of the same region. Sequential recording of the same ciliated cells before and after addition of 8-Br-cGMP to the trachea explant. As in Fig 9, 8-Br-cGMP does not change the orientation of the beating of the cilia in cells with uncoordinated ciliary beat. Download video

Importantly, the sequential recording setup allowed us to follow the ciliary beating pattern and frequency in individual cells (Fig 8M–O), demonstrating that within seconds after addition, 8-Br-cGMP affected the CBF in most of the individual ciliated cells: increasing the CBF in cells with cilia that beat in apparent coordination with the neighboring cells; increasing the CBF in cells in which the beating was uncoordinated with that of the neighbors; and converting immotile cilia into motile ones. However, we did not observe visible changes in the cilia beating direction: in cells in which the ciliary beating lacked coordination with that of the neighboring cells, cilia continued beating in an uncoordinated manner upon addition of 8-Br-cGMP, albeit with higher CBF (Video 7).

Remarkably, even in the presence of uncoordinated ciliary beating, the flow dynamics parameters were improved for the entire recorded region. The rescuing effect of 8-Br-cGMP on the flow in nNOS KO mutants was commensurate with the severity of the ciliary polarity and ciliary beating uncoordination defects: in the regions of the trachea where the defects were moderate and not more than half of all ciliated cells were beating without coordination with their neighbors, addition of 8-Br-cGMP improved the flow velocity despite the presence of cells whose cilia beat without coordination. However, in the regions with severe defects (with most cells beating without coordination) treatment with 8-Br-cGMP was not able to rescue the flow velocity despite the increased CBF. Together, these data suggested that the 8-Br-cGMP-induced increase in the fluid flow velocity was mainly due to the increase in CBF.

### Loss of nNOS disrupts the rotational polarity of basal bodies

Impaired coordination of the ciliary beating in the nNOS KO trachea implies the presence of defects in rotational polarity of the cilia. The rotational polarity of ciliated cells is largely mediated by microtubules, which connect the basal feet into a joint network, thus enabling the concerted rotation of basal bodies in the direction of the flow (Werner et al, 2011; Kunimoto et al, 2012; Vladar et al, 2012). Because nNOS was observed in association with microtubules and with the basal feet (Fig 1L), we sought to determine whether nNOS might contribute to the establishment of the cortical microtubule network.

To evaluate the input of nNOS to the rotational polarity of the tracheal ciliated cells, we selected a pair of markers: Chibby1 (Cby1), whose presence marks the transition zone of cilia (Burke et al, 2014), and ODF2, a marker of basal feet (Kunimoto et al, 2012). SIM visualization of these markers allowed for reliable reporting of both the rotational polarity and the spacing pattern of the basal bodies. The vectors, connecting the Cby1 and ODF2 puncta for each basal body in wild-type tracheal ciliated cells, pointed in a similar direction toward the larynx, thus demonstrating a clear pattern of rotational polarity within both single cells and groups of neighboring cells (Fig 9A and B). Moreover, the Cby1/ODF2 markers revealed that the basal bodies of tracheal ciliated cells were organized in parallel rows oriented perpendicularly to the median vector of rotational polarity (Fig 9A–D). This distinct orientation of the basal bodies’ rows was shared by neighboring cells (Fig 9A and C). This observation, made with the tracheal tissue, echoed the report of stereotypic row-like arrangement of the basal bodies in the ciliated tracheal cells grown as air-liquid interface (ALI) cultures (Herawati et al, 2016).

In contrast to the results for the wild-type tracheas, the pattern of rotational polarity, as visualized by Cby1/ODF2, was significantly distorted in the nNOS mutants (Fig 9E–G). In the areas with a mild distortion phenotype, coordinated rotational polarity was observed within single ciliated cells; however, the median vectors of rotational polarity were poorly coordinated between neighboring cells (Fig 9E), thus indicating that in the absence of nNOS, the cilia of the neighboring cells may be poised to beat against each other.

In the cell areas with a more severe distortion phenotype, the pattern of rotational polarity of the basal bodies was scrambled even within single cells (Fig 9F); notably, the spacing pattern in those cells was also distorted, and no apparent rows of basal bodies were observed. Because the orientation of ciliary beating is not coordinated, cilia may be poised to beat against each other even within the same cell, thus interfering with the task of directional flow generation.

SIM images of the patterns of the basal feet-microtubules connections revealed that in the wild-type ciliated cells, the microtubules (marked by α-tubulin) were attached to the most basal aspects of the basal feet (marked by ODF2) and formed a dense network (Fig 9H and I). In the wild-type ciliated cells, each basal foot was connected with the microtubules (Fig 9I, J, and M). In contrast, in the nNOS KO cells, only 75% of the basal feet were connected to the microtubules (Fig 9K, L, and M). Moreover, in the wild-type cells, a consistent overlap of the signals for ODF2 and α-tubulin was observed, with a steady Pearson correlation coefficient (PCC) of 0.72 ± 0.1, thus suggesting proximity between the basal feet and microtubules; in contrast, in the nNOS KO, the PCC varied significantly between individual basal feet, ranging from the wild-type–like value of 0.65 to 0.01, a value indicating a lack of significant signal overlap.

TEM images of nNOS KO ciliated cells (Fig 9N and O) showed a sparse microtubule network and basal feet lacking connections with the microtubules and thereby being excluded from the network. This type of defect suggested that nNOS and its association with basal feet and microtubules are essential for the formation of a properly formed and dense network connecting basal bodies, thereby enabling their cooperative rotation in the direction of the flow.

### Loss of nNOS disrupts the polarity of the actin cytoskeleton in ciliated cells

**Figure 10.**
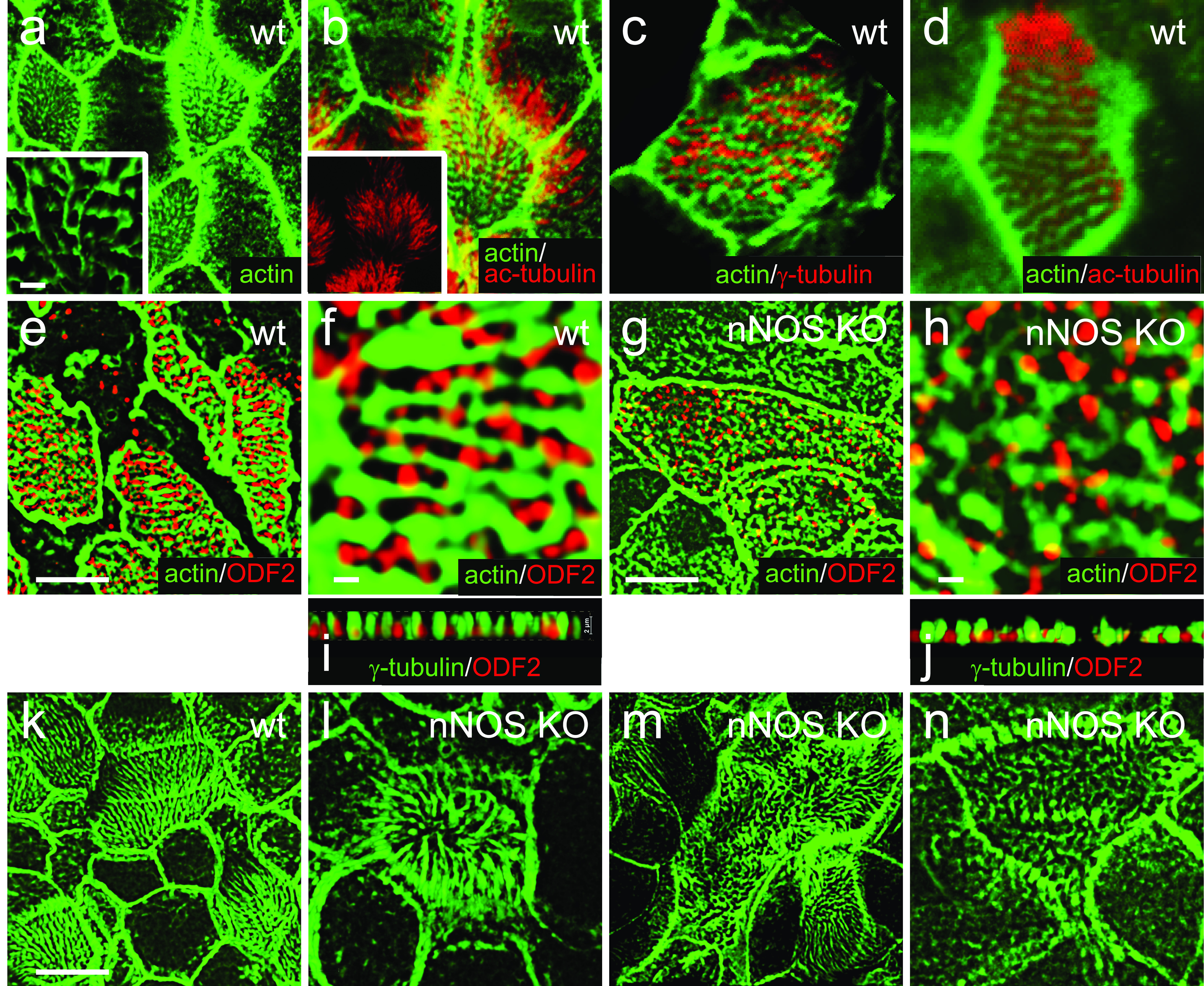
Polarity of the apical actin grid is related to the spacing pattern of the basal bodies. **(A)** Apical actin grid displays a windowpane pattern with opening for cilia. Inset shows a magnified region of 3D reconstruction, demonstrating rows and columns formed by the actin grid; note that the longitudinally oriented actin cables are running slightly more apically (above) than the crossing them cables. **(A, B, C, D)** Scale bars are 1 μm in (A, B, C, D). **(B)** Axonemes are oriented along the longitudinally oriented actin cables. Inset shows the cilia axonemes alone. **(C)** Basal bodies (labeled by γ-tubulin) are positioned in rows separated by actin cables of the actin grid. **(D)** An optic section at the base of cilia shows cilia (labeled by acetylated α-tubulin) positioned in rows, separated by actin grid cables. **(E, F, G, H, I)** Rows of basal bodies (labeled by ODF2) in trachea are attached to the actin cytoskeleton of the ciliated cells; such geometry of the apical actin grid provides a template for the spacing pattern of the basal bodies. **(G, H, I, J)** Apical actin grid in the ciliated cells of the nNOS KO has more chaotic structure because of shorter and poorly oriented actin cables. Likewise, the basal bodies, still attached to the actin cortex, are also chaotically organized by following the distorted shape of the actin grid template. **(K, L, M, N)** The range of distortion of the regular organization of apical actin grid in nNOS KO ciliated cells (L, M, N), compared with the windowpane pattern in the wild type (K).

Our results show that in ciliated cells of the wild-type trachea, a cortical actin network forms a typical pattern of a windowpane-shaped grid with openings for individual cilia (Figs 1 and 10A–F, I, and K). This grid, formed by intersecting actin cables, was polarized: in each ciliated cell, one set of the actin cables was aligned with the rows of the basal bodies, whereas the other set of the cables ran orthogonally, in the direction of the flow and ciliary orientation, with individual basal bodies positioned in the openings of the actin grid (Fig 10F and I). The polarity of the actin grid was shared by neighboring ciliated cells, thus manifesting tissue-level polarity (Fig 10A and E). Because the basal bodies of cilia are attached to the apical actin cortex after docking (Werner et al, 2011; Vladar et al, 2012; Herawati et al, 2016), the apical actin grid thereby provides a template, imposing its distinct geometry on the spacing pattern of the apically docked basal bodies.

In contrast to the regular pattern of the apical actin cytoskeleton observed in the wild-type trachea, the windowpane geometry of the apical actin cytoskeleton in nNOS KO mice was noticeably distorted, frequently showing disordered shapes of the actin grid and the loss of coordination in the geometry and orientation between neighboring ciliated cells (Fig 10G–J and L–N).

In the nNOS KO ciliated cells, basal bodies remained attached to the distorted apical actin cytoskeleton (Fig 10H and J). The typical spacing pattern of basal bodies in rows and the tissue level polarity of ciliary spacing patterns between neighboring ciliated cells deteriorated in parallel to the degree of distortion of the actin cortex in the mutant cells, essentially disappearing in cells with highly distorted actin cortex (Fig 10L–N). Therefore, the distortion of the geometry of the apical actin cytoskeleton in nNOS KO ciliated cells defined the distortion of the spacing pattern of the basal bodies observed in the mutant cells.

### nNOS and RhoA mediate establishment of the pattern of the apical actin cytoskeleton in ciliated cells

We next asked whether nNOS may control the proper arrangement of the apical actin grid by modulating relevant signals and effectors that control formation of the actin cables. RhoA, a major regulator of actin polymerization dynamics, is active in numerous cell polarity contexts (Besson et al, 2004; Pan et al, 2007; Zaoui et al, 2008; Miller & Bement, 2009). Importantly, in multiciliated epithelial cells, RhoA activity is essential for organizing the apical actin cytoskeleton during ciliogenesis (Pan et al, 2007) and is crucial for the anchoring of basal bodies to the apical domain (Sedzinski et al, 2017).

**Figure 11.**
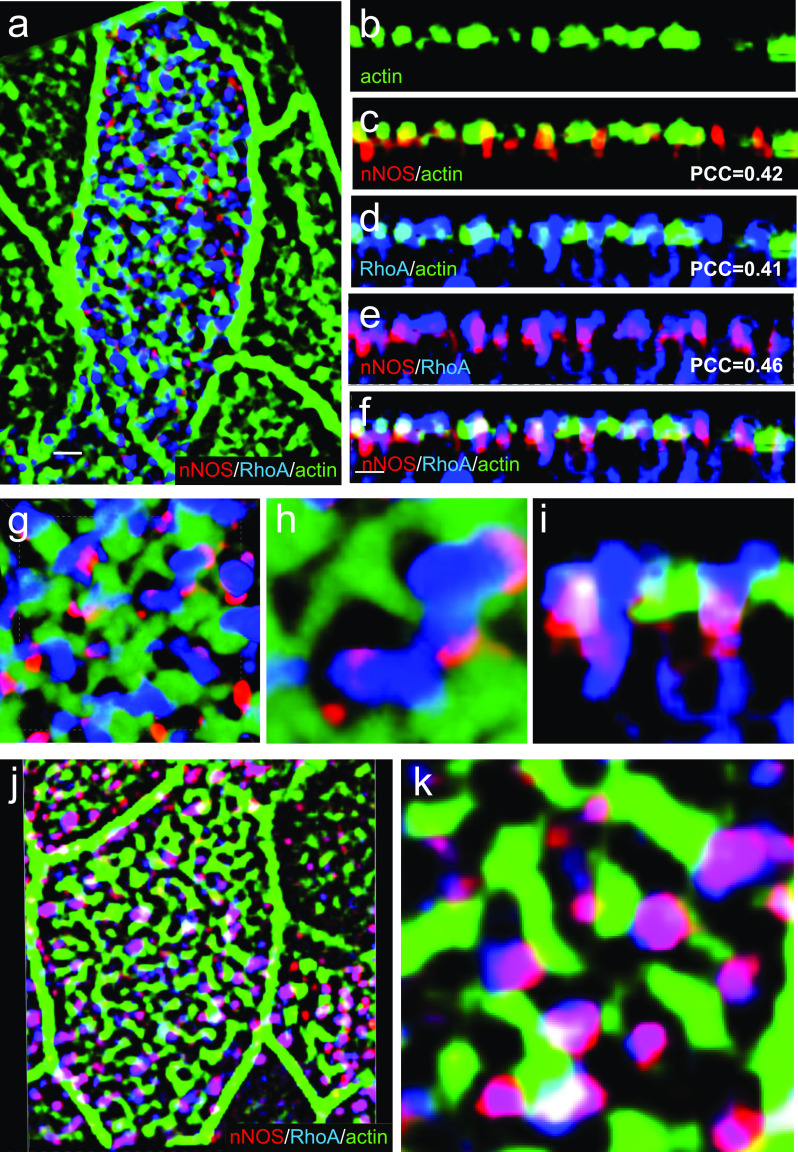
Active RhoA is associated with nNOS. **(A)** Ciliated cell in the wild-type trachea, labeled for actin, nNOS, and active form of RhoA. Scale bar is 1 μm. **(B, C, D, E, F)** Z-sections of (A): active RhoA is associated with nNOS, their signals overlapping with Pearson correlation coefficient = 0.46. Scale bar is 2 μm. **(A, G, H, I)** 2.5× (G) and 7× (H, I) magnifications of (A), with Z-section of (H) shown in (I). Images in (B, C, D, E, F, G, H, I) show that nNOS is positioned in the openings of the apical actin grid and is associated there with the basal bodies (also see Fig 1E and F). **(J, K)** The pattern of association of nNOS and active RhoA revealed in the trachea is also observed in air-liquid interface cultures of the tracheal ciliated cells.

**Figure 12.**
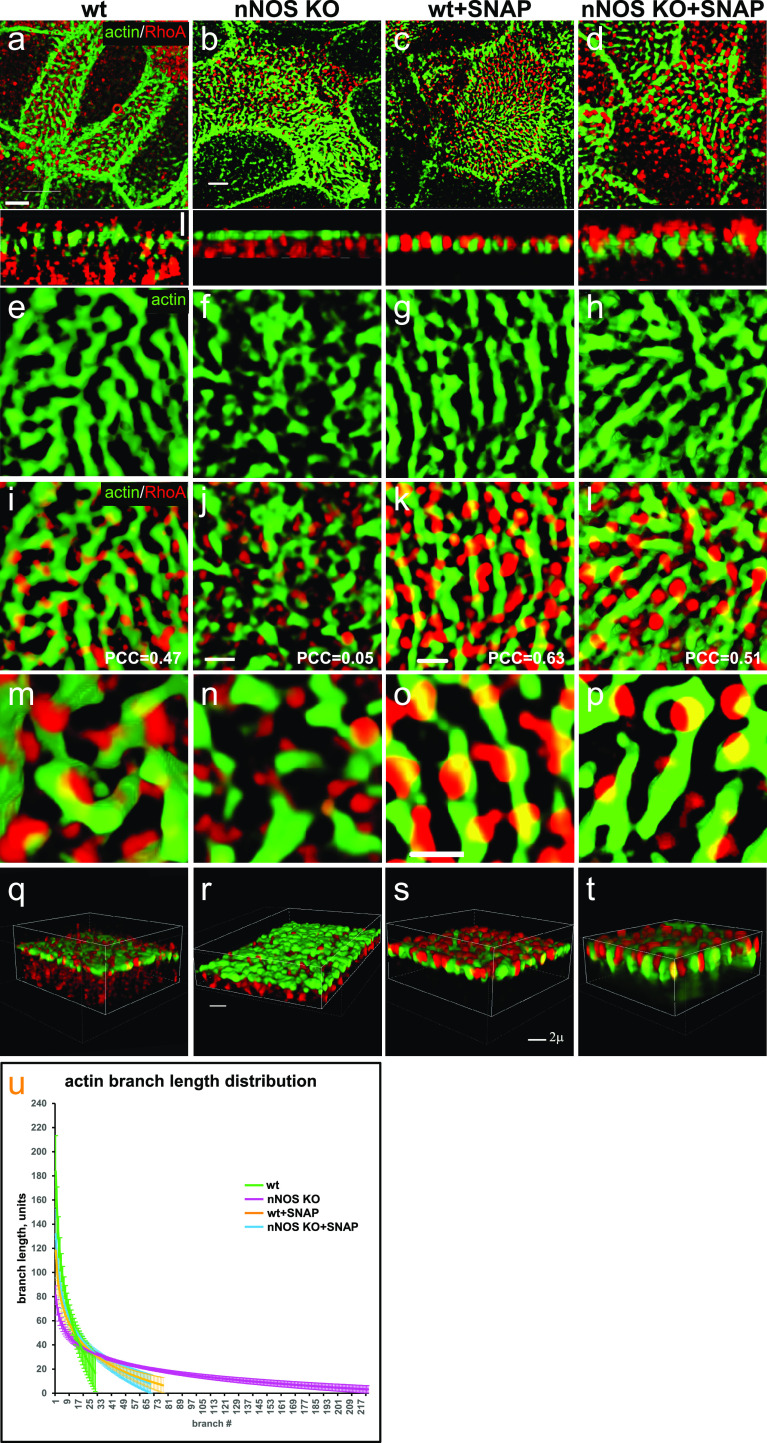
nNOS/Nitric oxide are essential for targeting the active RhoA to the apical actin cortex. Treatment with nitric oxide donor SNAP increases loading of actin with active RhoA and is associated with increased length of the actin cables forming the apical actin grid. **(A, B, E, F, I, J, M, N)** Distortion of the structure of apical actin cortex (the actin grid) in the ciliated cells of the nNOS KO (B, F), in comparison with the wild type (A, E), trachea. In the wild type, active RhoA is present both within the apical actin grid (where it is concentrated in the openings for the basal bodies, where nNOS is also found) and in the cytoplasm just below the actin cortex (Z-section in A). Deletion of nNOS resulted in the loss of RhoA activity within the apical actin cortex and its accumulation in the cytoplasm below the apical actin network (B). **(C, D, G, H, K, L, O, P)** Addition of SNAP for 30 min to the wild-type or nNOS KO tracheal explants leads to the translocation of RhoA activity from the cytoplasm to the apical cortex and augmentation of RhoA association with actin (restored Pearson correlation coefficient) at the expense of the fraction of RhoA in the cytoplasm (Z-sections). **(Q, R, S, T)** 3D reconstruction of the SIM stack images. **(U)** Changes in the distribution of actin branch lengths in nNOS KO apical cortex (increased presentation of the short branches) and their alleviation upon addition of SNAP (distribution of the actin branch lengths shifts towards longer branches). Scale bars are 2 μm in (A, B, C, D), 5 μm in (E, F, G, H, I, J, K, L), and 10 μm in (M, N, O, P).

Thus, Rho A was deemed an attractive candidate to test for the possible interaction of nNOS with potent regulators of actin polymerization. Using antibodies specific for the active GTP-bound form of RhoA (Benink & Bement, 2005), we found the active RhoA signal in close proximity to nNOS (PCC = 0.46) in the openings of the apical actin cortex of ciliated cells in the trachea (Fig 11A–I); we also found a similar association between RhoA and nNOS (with a higher PCC of 0.7) in the apical actin cortex of re-differentiated mouse trachea ciliated cells grown in ALI cultures (Figs 11 and 12). Besides the apical cortex-bound active RhoA, a substantial fraction of active RhoA was also detected in the ciliated cells’ cytoplasm just beneath the actin cortex (Fig 11D–F).

Given that the nNOS signal marks the positions of basal bodies in the openings of the apical actin grid (Fig 1E and F) and is found near active RhoA, we asked whether nNOS/NO signaling might be important for RhoA activity and polymerization of the cortical actin, and consequently for basal body attachment to the actin cortex and their spacing pattern.

We found that deletion of nNOS resulted in a significant loss of active RhoA associated with the apical actin in the tracheal ciliated cells, with a concomitant increase in the fraction of RhoA localized in the cell cytoplasm (Fig 11A–D). The loss of RhoA from the actin grid was reflected in a significantly decreased PCC for RhoA/actin association (from 0.47 in the wild type to 0.05 in the mutant Figs 11 and 12. The diminished presence of active RhoA at the apical actin grid of the mutants was associated with a more disordered grid structure and a shift in the distribution of actin filaments forming the grid toward significantly shorter lengths compared with the wild type (Fig 12U). Importantly, when examining both wild-type and nNOS KO tracheal preparations 30 min after the addition of the NO-releasing donor SNAP we detected increased presence of active RhoA in the apical cortex at the expense of its presence in the cytoplasm (Fig 12A–D and Q–T); an increased association between active RhoA and apical actin (from 0.05 to 0.51 PCC) (Fig 12I–T); and a shift in the length distribution of the actin polymers forming the actin cortex toward longer filaments (Fig 12E–P and U).

We concluded that nNOS/NO is necessary for directing RhoA activity to the apical actin cortex where RhoA acts as a positive regulator of actin polymerization, essential for the formation of longer actin filaments and an organized structure of the actin grid, thereby directing proper spacing of the basal bodies. Notably, despite defects in the organization and geometry of the apical actin grid when nNOS is absent, the basal bodies remained attached to the apical cortex; this result suggested that nNOS, whereas being necessary for the structure of the actin grid, is dispensable for basal bodies’ attachment to the apical actin.

### nNOS is associated with proteins of the Par3 complex in the axonemes and is important for their axonemal localization

Proteins of the Par3 complex, including aPKC, are active in numerous contexts involving establishment of apical cell polarity; in addition, these proteins have been implicated in ciliogenesis (Fan et al, 2004; Pruliere et al, 2011; Chen & Zhang, 2013; Hong, 2018). Therefore, we examined the potential connections among Par3, aPKC, RhoA, and nNOS in tracheal ciliated cells.

**Figure 13.**
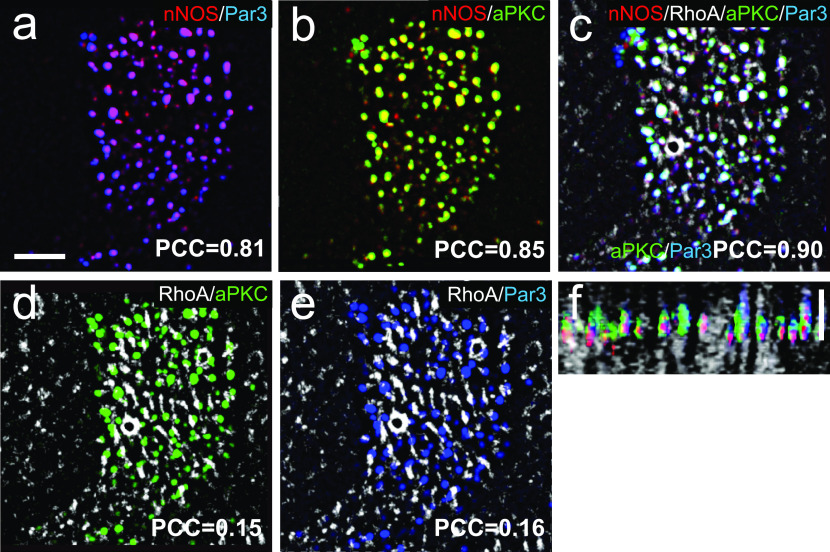
nNOS is associated with RhoA and proteins of the Par3 complex in different cilia domains. **(A, B, C)** nNOS is associated with Pa3 and aPKC with high Pearson correlation coefficient (PCC) of 0.81 and 0.85, correspondingly, and aPKC and Par3 are colocalized (PCC = 0.9). **(A, B, C)** Scale bar is 2 μm in (A, B, C). **(D, E)** Par3 and aPKC are poorly associated with RhoA, with PCC = 0.15 and 0.16, correspondingly. **(E)** 3D reconstruction of the Z stalk indicates heterogeneity in the distribution of examined markers (nNOS, RhoA, Par3, and aPKC) along the Z aspect of the basal area of the cilia image.

**Figure 14.**
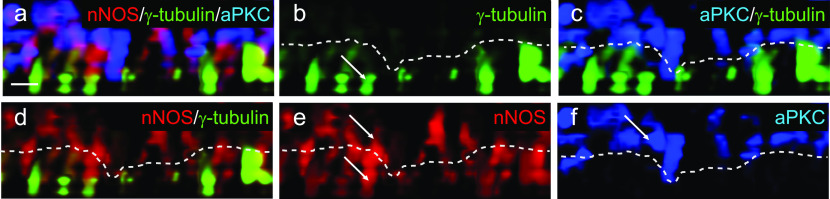
aPKC is expressed within the axoneme region of cilia. **(A, B, C, D, E, F)** whole mount immunohistochemistry of wild-type tracheal explants with markers for aPKC, nNOS, and basal bodies (γ-tubulin). nNOS is present both in the basal bodies and the axoneme domains (A, B, D, E). **(A, C, F)** aPKC overlaps with nNOS in the axoneme domain (A) but not with the basal bodies (A, C, F).

**Figure 15.**
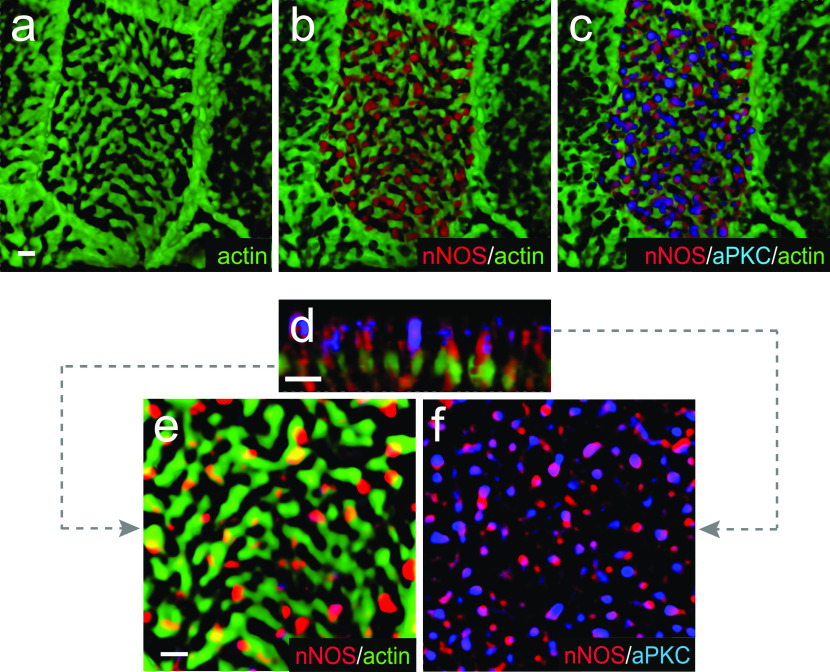
aPKC and nNOS signals overlap in the axoneme region of the ciliated cells. **(A, B, C, D, E, F)** Whole-mount immunohistochemistry of wild-type tracheal explants. Immunostaining for nNOS, aPKC, and actin (A, B, C, D, E, F). **(D, E, F)** correspond to different levels indicated by dotted lines on the Z-section (D). nNOS and PKC signals associate at the level of axonemes but not the apical actin grid and basal bodies.

Immunohistochemical analysis and SIM imaging of the apical area of ciliated cells showed high degree of overlap between nNOS and Par3, nNOS and aPKC, and Par3 and aPKC (PCC 0.81–0.90; Fig 13A–C and F). However, both the Par3 and aPKC signals showed only low overlap with the signal of active RhoA (Fig 13D–F). To determine the specific domain of ciliated cells’ apical region where Par3 and aPKC might interact with nNOS, we examined the localization of these proteins in relation to the basal bodies (Fig 14). The nNOS signal extended apically beyond the basal bodies into the area of proximal axonemes, where it colocalized with aPKC (and, by proxy, with Par3) (Fig 14). However, the aPKC signal, unlike that of nNOS, did not extend into and below the apical membrane, into the region of the basal bodies, where the nNOS signal overlaps with that of γ-tubulin (Fig 14A–F), or into the apical actin cortex area where nNOS is associated with RhoA (Fig 15A–F). These results suggest that nNOS closely associates with the Par3 complex proteins only in the axonemal domain.

**Figure 16.**
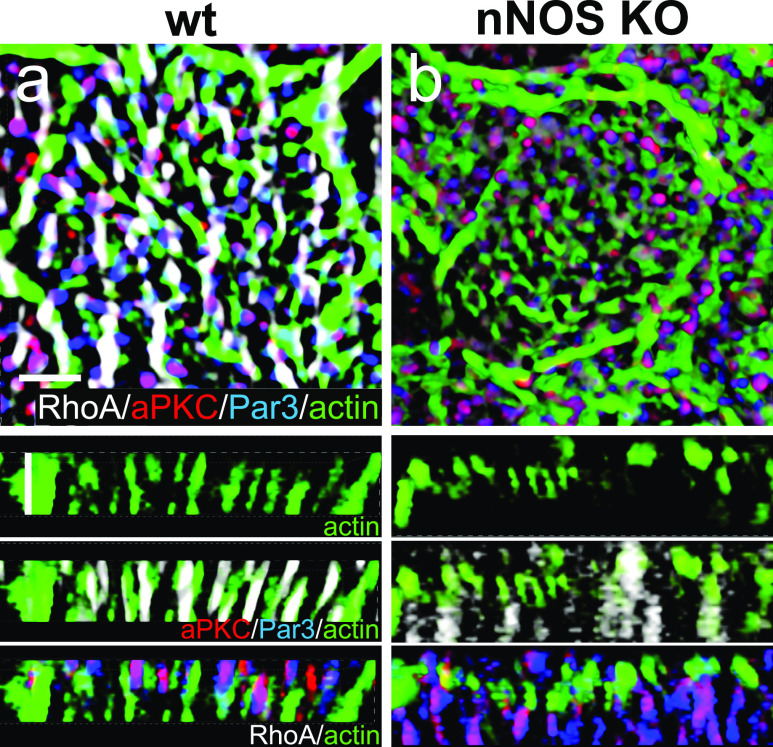
Deletion of nNOS causes loss of the proteins of Par3 complex. **(A, B)** Whole mount immunohistochemistry of wild-type and nNOS KO trachea explants with markers of RhoA, Par3, aPKC, and actin. **(B)** In nNOS KO, where the distortion of the regular structure of the cortical actin grid and the loss of RhoA are evident (B), there is also loss of Par3 and aPKC, which are associated with nNOS in the wild-type axonemes. **(B)** aPKC and Par3, whereas remaining colocalized, are significantly diminished in the axonemes and are present in the cytoplasm below the actin cortex (B). Horizontal and vertical scale bars are 2 μm in (A, B).

We next examined the distribution of the Par3 and RhoA proteins in ciliated cells of the nNOS KO (Fig 16A and B) and found that substantial fractions of Par3 and aPKC were lost from the proximal axonemes and instead appeared mostly below the actin cortex. Notably, the association between Par3 and aPKC remained intact (PCC = 0.85), thereby suggesting that nNOS is essential for the targeting of Par3 and aPKC protein complexes to the axonemes but is dispensable for the Par3/aPKC association.

### nNOS and ciliary maturation

Basal body docking is critical for ciliary growth and is dependent on RhoA activity and the proper assembly of the cortical actin network (Sedzinski et al, 2017). The ensuing maturation of the cilia after docking requires the concerted action of several other proteins involved in the assembly of the transition zone, a specialized domain of cilia that establishes reliable transport of proteins and other required components between the cell cytoplasm and the growing axoneme (Avidor-Reiss et al, 2017).

Although we did not observe changes in the ciliary docking in the nNOS mutant (Fig 2M–P), we detected abundant ciliary growth defects, which manifested as sparse and short cilia (Fig 2C–E), thus indicating potential defects in cilia maturation. The Cby1 protein, localized in the transition zone of the cilia (Burke et al, 2014; Siller et al, 2015), is critically involved both in the docking of the basal bodies to the apical cell membrane and in the maturation of cilia. The number of correctly formed cilia is diminished in Cby1 mutants because most of the basal bodies failed to migrate from the cytoplasm and dock apically, whereas the cilia whose basal bodies successfully dock are sparse and very short (Voronina et al, 2009; Love et al, 2010; Li et al 2015, 2016; Siller et al, 2015). The latter phenotype resembles that of the ciliated cells of the nNOS KO mutants in which we detected docked basal bodies and rudimentary short axonemes (Fig 2P).

**Figure 17.**
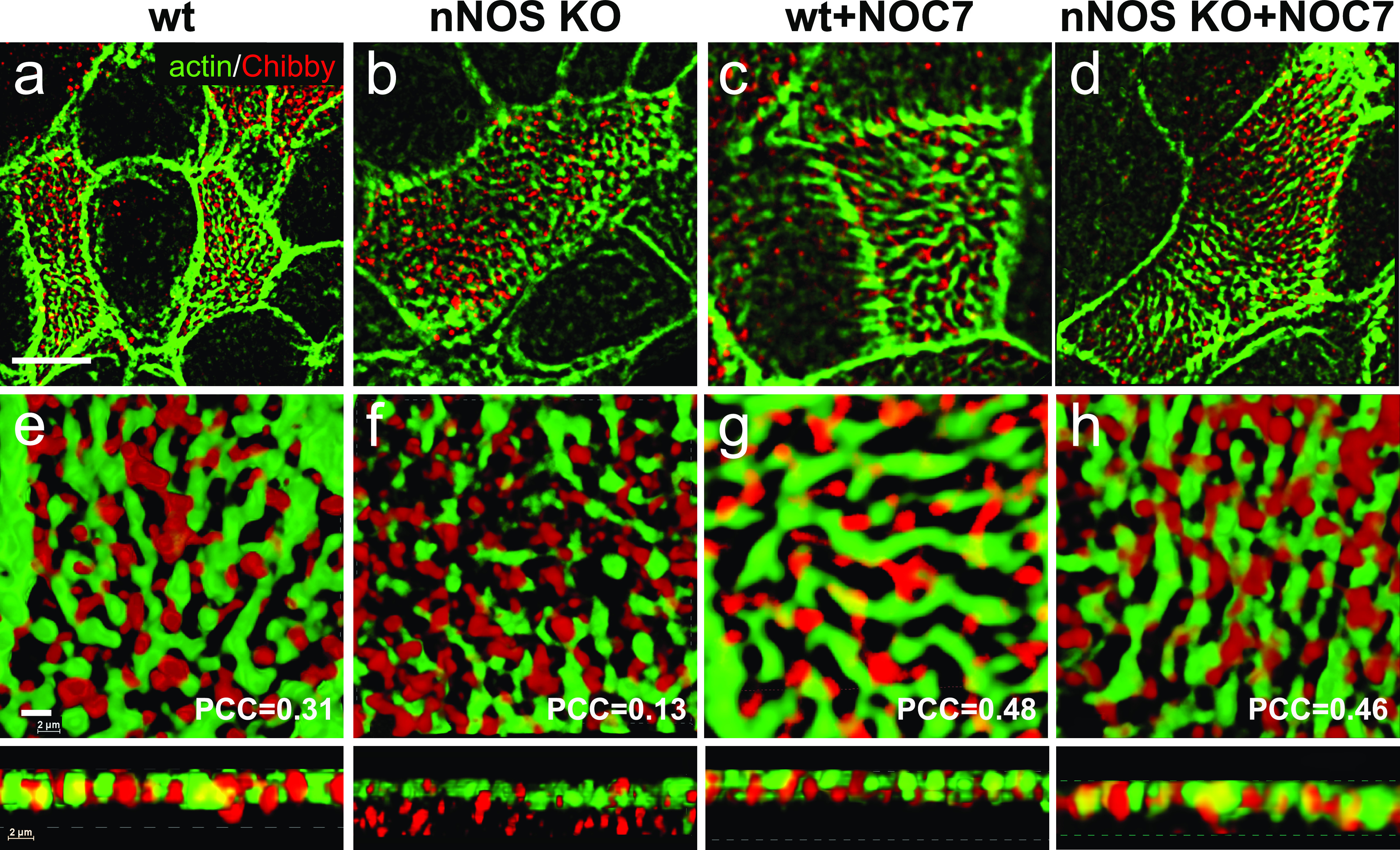
Position of Cby1 in the transition zone of the cilia is dependent on nitric oxide. **(A, B, C, D, E)** Cby1, a protein crucial for the assembly of mature cilia docked to the apical cell cortex, is detected in the openings of the apical actin grid in the ciliated cells of the wild-type trachea. **(B, C, D, E, F)** In the ciliated cells of the nNOS KO trachea, a significant fraction of Cby1 is not associated with the actin grid and remains in the cytoplasm below the cortex. **(C, D, E, F, G, H)** Treatment with nitric oxide donor NOC7 for 30 min augments association of Cby1 with the actin cortex in the wild-type trachea and leads to translocation of Cby1 from the cytoplasm in the nNOS KO, rescuing the association of the protein with the actin cortex. Scale bar is 5 μm in (A, B, C, D) and 2 μm in (E, F, G, H).

As expected, in the wild-type ciliated cells a substantial fraction of the Cby1 protein was present at the apical domain and was associated with the apical actin grid (Fig 17A and E; PCC = 0.31). In contrast, in the nNOS KO a large fraction of Cby1 did not overlap with the apical actin cortex and instead remained just below the cortex, within a micrometer distance (Fig 17B and F), and the actin/Cby1 PCC decreased to 0.13. We then asked whether mislocalization of Cby1 in the nNOS mutant might be rescued by the addition of exogenous NO; indeed, treatment of the tracheal explants with NOC7 significantly alleviated this defect and the Cby1 signal was again associated with the apical actin cortex (PCC of 0.48 and 0.46 for the wild type and nNOS KO, respectively; Fig 17C–H).

These results demonstrate that the association between Cby1 and apical actin, which is normally characteristic of the docked state of basal bodies, is dependent on nNOS and that nNOS/NO might be required for the final step of association of Cby1 with the basal bodies and actin cortex, a step necessary for the generation and maintenance of ciliary axonemes.

## Discussion

Generation of mucus flow by multiciliated cells enables airway clearance. This process requires the precise coordination of several cellular functions, including the properly established planar polarity of ciliated cells and robust beating of the cilia. Numerous cilia must be oriented in the direction of the flow and must also be arranged on the cell surface in a pattern enabling their effective cooperative movements. Moreover, groups of adjacent cells must coordinate their ciliary beat periodicity and beat in unison to achieve efficient propulsion of the mucus along the airway. The mechanisms supporting this concerted activity of the tracheal ciliated cells are poorly understood.

Our results point to nNOS-produced NO as an important regulator of ciliary activity in the trachea, capable of integrating key modalities that determine the generation of efficient flow. These findings also indicate that insufficient NO bioavailability compromises the activity of ciliated cells and the generation of directional flow and may thus contribute to ciliopathies. These roles of nNOS/NO are supported by several lines of evidence in our study.

First, we found that in the ciliated cells of the mouse trachea nNOS is distributed in a pattern that allows its interactions with different cellular domains: the basal body, the apical actin and microtubules cytoskeletons, and factors of the transition zone and the axoneme of the cilia.

Second, we show that nNOS/NO is involved in distinct aspects of the planar polarity of the ciliated cells in the trachea: the initial patterning of the mucociliary epithelium, executed by the PCP pathway; the setting of the stereotypic spacing pattern of the basal bodies and cilia, defined by the formation of the apical actin grid; and the rotational polarity of the cilia, determined by the interactions between the basal feet and the microtubules network. Some of these features, when compromised by nNOS loss, can be efficiently mitigated by the addition of exogenous NO.

Third, we demonstrate that nNOS/NO regulates the efficiency of the ciliary beat in the mouse trachea. We also show that the ability of NO to support CBF is mediated by the nNOS-NO-sGC-cGMP pathway. These conclusions are supported by the finding that the sGC inhibitor ODQ suppressed CBF, whereas the cGMP analog increased the CBF; moreover, in the absence of nNOS, the cGMP analog can act similarly to exogenous NO-releasing compounds and fully rescue the ciliary beat and restore the flow in the trachea.

Yet another factor that might potentially be affected by NO, is the mucus itself–both its composition and the neural control of its secretion (Boucher, 2019). Intriguingly, NO has been reported to affect both aspects, and determining whether nNOS/NO acts as a multipronged regulator of the mucociliary transport in the airway should prove interesting.

Our new observations were largely enabled by the techniques that we developed, which allowed for concurrent analysis of the flow and CBF. This capability greatly increased the reliability of the analysis and permitted direct comparison of treatments effects across various preparations. A further modification of this technique, sequential recording of the same cells upon a particular treatment in a perfusion flow camera, allows for even more accurate analysis of the ciliary activity: first, it enables prolonged recordings of the ciliary activity without the rapid decay of CBF (that is inevitable when trachea specimens are placed in sealed viewing chambers), and second, by focusing on the responses of individual cells rather than applying conventional averaging of the responses across different specimens and regions of interest.

Beyond the findings described above, these techniques revealed an intriguing feature of the efficient directional flow in the trachea: – a possible link between the rate of the ciliary beat and the coordination of the beat across multiple cilia and multiple cells. We found that coordination of the ciliary beat in wild-type trachea rapidly deteriorated when CBF decreased after its exposure to ODQ; at the same time, the treatment did not affect the planarity. Conversely, exposure of the nNOS KO trachea to NO donors or 8-Br-cGMP, which increased the CBF without significantly affecting the planar polarity of the ciliated cells, also increased the coordination of the ciliary beat in individual cells and groups of adjacent cells, and improved the flow across the tissue.

The increase in flow velocity in these experiments occurred despite the presence of mutant cells with greatly distorted planar polarity and cilia that continued to beat out of synchrony with their neighbors. These results raise a possibility that a robust ciliary beat might also support the coordination of the ciliary beat in individual ciliated cells and across groups of cells in the trachea, even under conditions in which a fraction of ciliated cells in a region show deficient polarization. It will be interesting to examine the link between the level of CBF and the coordination of the ciliary beat, and determine whether NO contributes to feedback or feedforward support of these two important modalities that eventually determine the efficiency of the airway clearance.

Mechanistically, our results provide strong evidence that the nNOS-NO-sGC-cGMP pathway mediates the action of NO on CBF. cGMP may activate cGMP-dependent kinase (PKG) and, potentially, cAMP-dependent protein kinase (PKA) (Lorenz et al, 2017), which in turn may phosphorylate dyneins or kinesins of the axoneme, altering the cilia beating. PKG has been detected in the axonemes of the tracheal cilia; moreover, in isolated cilia preparations submicromolar concentrations of cGMP induced phosphorylation of the ciliary proteins (Gertsberg et al, 2004; Wyatt, 2015).

The nNOS-NO-sGC-cGMP pathway might be less important in the control of planar polarity by nNOS/NO than the control of CBF. Our results indicate that nNOS might instead rely on RhoA as a possible effector of the actin polymerization and consequently the actin grid organization and the resulting spacing pattern of the basal bodies and cilia. The distribution of nNOS along the basal body allows it to associate with the actin filaments at the sites of basal body attachment to the apical actin cortex and to regulate the activity of RhoA molecules which are present apically, both within the actin cortex and in the cytoplasm just beneath the cortex. Active RhoA, a major regulator of actin polymerization, is positioned at the sites of basal body attachment to the cortical actin grid, within the grid openings. Our data are compatible with a model in which nNOS/NO recruits activated RhoA to the apical actin cortex where RhoA enhances the polymerization dynamics of actin in the apical cortex surrounding the basal bodies. By promoting the formation of actin filaments of optimal length, RhoA may therefore determine the formation of a structured template for the spacing pattern of the basal bodies. nNOS appears to regulate the transition of the active form of RhoA from the cytoplasm and its association with the grid and promotion of actin polymerization in the grid. nNOS/NO may control this cytoplasm-apical cortex transition of active RhoA by negatively regulating RhoA-GDP dissociation inhibitors, or by positively regulating the RhoA guanine nucleotide exchange factors or RhoA farnesylation and the membrane insertion of the farnesylated RhoA molecules.

nNOS is also necessary for the apical distribution of Par3, aPKC, and Cby1, which were present in the proximal axonemes in the wild-type cells, but mostly accumulated under the apical actin cortex in the mutant trachea. These deficits may be related to the absent or very short cilia observed in the nNOS knockout (Figs 13–17). Commonly, a decrease in the number of cilia is associated with the defects of docking of the basal bodies to the apical membrane (Park et al, 2008; Voronina et al, 2009; Burke et al, 2014); however, our TEM images do not indicate failed docking of the basal bodies (Fig 2N and P). The loss of Cby1, Par3, and aPKC in the axonemes of the nNOS mutant might compromise the maturation and growth of the axonemes, resulting in extremely short cilia, as observed in our SIM and TEM images. This would effectively lead to a phenotype of sparse cilia that we observe, even with the basal bodies docked to the membrane.

Absence of nNOS is also associated with defects in the planar polarity of Vangl1 distribution (Fig 3). These defects suggest that nNOS/NO signaling interacts with the PCP pathway which is critical for establishing proper tissue polarity, a conclusion consistent with the previously discovered link between NO and Dsh signaling pathways during *Xenopus* embryogenesis (Peunova et al, 2007).

We have fewer insights at this moment regarding the potential mechanisms of the interaction of nNOS with the apical network of microtubules that connects the basal feet of the cilia and enables the cooperative rotation of numerous basal bodies, thereby orienting individual cilia in the direction of the flow (Vladar et al 2009, 2012, 2016; Werner et al, 2011). A less dense network of microtubules and a weaker association of the basal feet with the microtubules may be at the core of the defects in impaired rotational polarity observed in nNOS KO mutants. These defects suggest distinct roles of nNOS/NO in the interaction of basal feet with microtubules. The overlap of nNOS and the basal foot marker ODF2 suggests that their interaction might be involved in the formation of the microtubule network connecting the cilia. Notably, the defects in rotational polarity in the nNOS mutants were not rescued by NO or cGMP, and whether this activity of NO might be inherently associated with the cGMP or RhoA pathways or might reflect a separate signaling branch, remains an open question.

Our model implies that nNOS-produced NO is capable of both regulating the fluid flow and rapid rearrangement of the cytoskeleton. Notably, the time scale of the rescue of the CBF and the flow by NO/cGMP, which occurred within seconds after the addition of the NO donors or 8-Br-cGMP, differs from the dynamics of the actin remodeling after adding NO donors to the trachea explants. We could detect the differences in the morphology of the apical actin cytoskeleton, the changes in distribution of RhoA activity within ciliated cells, and its transition from the cytoplasm toward the apical domain within 30 min after exposure to NO donors. This suggests that molecular pathways employed by NO/cGMP in control of CBF may encompass factors and reactions different from those that are engaged in the apical actin remodeling. The involvement of nNOS in multiple molecular signaling pathways essential for the ciliated cell function is also supported by the localization of nNOS in different domains of the ciliated cells and its local association with different proteins partners: apically with RhoA and actin, and in the cilia axonemes with Par3 and aPKC.

Pertinent to the notion of NO as a regulator of rapid cytoskeleton rearrangement, there are numerous examples of fast cytoskeleton reorganization in NO-dependent physiological responses. This includes the response of constricted blood vessels to NO-releasing drugs (opening of the vessel lumen and decreasing the blood pressure); nNOS-mediated penile erection; or bladder sphincter opening in response to the urine pressure (Balligand et al, 2009; Hurt et al, 2012). These NO-mediated processes occur on the time scale ranging from minutes to hours, attesting to the capacity of the NO signals for rapid action. Notably, the rearrangement of basal bodies in cultured ciliated cells was also found to be a relatively fast process. Echoing the results of our experiments, chaotic basal bodies distribution, distorted by inhibition of actin polymerization, quickly returned to the polarized arrangement in rows upon the washout of the actin polymerization inhibitor (Herawati et al, 2016).

In sum, our results highlight nNOS and NO as factors that allow for close integration of two processes that govern the efficient mucociliary clearance in the trachea: proper polarization of ciliated cells and efficient beating of the cilia. These two activities of nNOS may be mediated by different signaling pathways–a RhoA-mediated pathway for polarity and a cGMP-mediated pathway for CBF.

In this investigation we demonstrated that in the multiciliated cells of the tracheal tube the rows of basal bodies and the orientation of cilia are set perpendicularly to each other and this pattern is defined by the polarity of the apical actin cytoskeleton. The role of the actin cytoskeleton in the spacing pattern of the basal bodies on the surface of the ciliated cells was recognized previously for the polarity of the ciliated cells in the embryonic skin of *Xenopus* (Werner et al, 2011) and the ciliated cells of the mouse trachea, isolated and grown as ALI cultures (Herawati et al, 2016). The pattern of the basal body rows that we found in the trachea explants resembles that of cultured ciliated cells (Herawati et al, 2016), indicating a robust control exerted over this feature.

Interestingly, when examining the brush-like arrangement of cilia in the trachea, we found that at the tissue-polarity level, the direction of the cilia beating was not fully aligned and parallel to the longitudinal axis of the trachea, but instead were set at an angle to this axis. This arrangement reflects the polarity of the apical actin grid, linked to the basal body spacing pattern, also being set at an angle to the longitudinal axis. Moreover, the direction of the flow (according to the tracking beads’ traces) was also most often oriented at an angle to the longitudinal tracheal axis. Although this arrangement appears somewhat counterintuitive because it does not appear to provide the shortest path to the mucus flow, similar observations have been made in live non-intubated animals, in which the orientation of the beads moving with the fluid flow was at an angle to the longitudinal axis of the trachea (Veres et al, 2017).

Our findings on the multiple tasks of nNOS/NO in ciliated cells may have broader implications for human ciliopathies. A decreased level of NO in exhaled gas is a rapid diagnostic test that allows for triaging of patients with chronic sinusitis, bronchiectasis, infertility, or other manifestations of PCD, to be followed by microscopic histological analysis to confirm or to rule out PCD (Walker et al 2012, 2013; Lobo et al, 2015; Werner et al, 2015; Horani et al, 2016; Knowles et al, 2016). Despite the importance of NO testing in clinical practice, the causal link between low NO levels and PCD so far remained obscure.

Our results provide a potential explanation for this connection. Note that while we here describe the effects of NO on CBF and support of robust fluid flow, it is also possible that flow in turn affects nNOS activity, perhaps through the activation of nNOS by the shear stress (Sato et al, 2008; Melikian et al, 2009; Hyndman et al, 2015; Zhang, 2016). Shear stress has also been shown to increase the CBF in the trachea (Tarran et al, 2006; Winters et al, 2007), which would lead to increased flow. We propose that NO, produced by shear stress-activated nNOS, through its ability to to increase CBF, may mediate a CBF-flow-shear stress-CBF feedback loop. This may imply that inefficient flow, impaired by various mutations that compromise cilia function (e.g., the numerous mutations identified as the cause of PCD and other ciliopathies) may also decrease stress-induced production of NO, thus further compromising the NO-mediated CBF/flow feedback loop. Therefore, diverse ciliopathy-causing mutations may lead to decreased NO, decreased CBF, and poor mucus flow in the airway, thereby explaining the correlation between low levels of exhaled NO and a range of ciliopathies.

Although PCD is a relatively rare disorder, affecting 1 in 25,000 people, our discovery of the mechanistic link between decreased NO availability and compromised flow and the restoration of the flow by a widely used NO donor may have broader implications. Insights from our model may serve as a gateway to the treatment of a broader class of ciliopathies and disorders of the airways of diverse genetic origin that may benefit from supplementation by NO.

## Materials and Methods

### Animals and cells

Mouse maintenance followed the guidelines for the use and treatment of laboratory animals from the National Institutes of Health and all procedures were approved by Animal Care and Use Committee of Stony Brook University. Mutant and wild-type animals were bred and housed at the animal facilities of Stony Brook University in a standard light- and temperature-controlled environment (12-h light/dark cycle; light on at 7:00 am; 21°C) with access to food and water ad libitum. Generation of nNOSKOex6 knockout mouse line (nNOS KO throughout the article) representing a null mutation of nNOS gene was described previously (Packer et al, 2003). This knockout line, generated by deletion of the active site encoded by exon 6 of the mouse gene, lacks functional mRNA or protein, and unlike the widely used nNOS mutant line (Cat. no. 2633; Jackson Labs), represents a true null mutant of the nNOS gene. In most experiments, the nNOS KO^+/+^ and nNOS KO^−/−^ mice from the same litter were used as wild-type and knockout pairs. Trachea specimens of nNOS-CreER::Ai9 transgenic mouse (Taniguchi et al, 2011) after induction of recombination with tamoxifen was a gift from Dr. Josh Huang (Cold Spring Harbor Laboratory). ALI cultures of ciliated cells of mouse trachea were developed and maintained as described previously (Li et al, 2016).

### Immunocytochemistry and microscopy

We used the following antibodies for immunocytochemistry: mouse monoclonal antibody to acetylated α-tubulin (T6793, dilution 1:1,000; Sigma-Aldrich); mouse monoclonal antibody to γ-tubulin (T6557, dilution 1:500; Sigma-Aldrich); mouse monoclonal antibody to α-tubulin (ABT 171, dilution 1:200; Millipore); goat polyclonal antibody to α-tubulin (2144S, dilution 1:200; Cell Signaling Technology); rabbit polyclonal antibody to cenexin (ODF-2) (HPA001874, dilution 1:500; Sigma-Aldrich); rabbit polyclonal antibody to aPKCζ (sc-937, dilution 1:500; Santa Cruz); rabbit polyclonal antibody to Vangl1 (HPA025235, dilution 1:500; Sigma-Aldrich); sheep polyclonal antibody to nNOS (ab6175, dilution 1:500; Abcam); rabbit polyclonal antibody to nNOS (ab76067, dilution 1:500; Abcam); rabbit polyclonal antibody to Par3 (07-330, dilution 1:200; Millipore); mouse monoclonal antibody recognizing the active form of RhoA (26904, dilution 1:200; West East Sciences); mouse monoclonal antibody to Chibby1 (dilution 1:100) generated by K-I Takemaru (Burke et al, 2014); chicken polyclonal antibody to rootletin (dilution 1:200), a gift from Dr. Tiansen Li, Massachusetts Eye and Ear Infirmary; and mouse monoclonal antibody to Na(+)/K(+) ATPase (Iowa Developmental Studies Hybridoma Bank). Secondary antibodies were anti-mouse, anti-rabbit, anti-sheep, or anti-goat antibodies conjugated to Alexa-488, Alexa-558, or Alexa-633 or Alexa 405 from Molecular Probes, used at dilution 1:500. Actin was visualized with phalloidin–Alexa-488 or phalloidin–Alexa-633 (Molecular Probes).

For whole mount immunocytochemistry staining freshly dissected trachea of wild-type or mutant animals were cut longitudinally, flattened by briefly placing the explants on filter paper with the cilia side up, and fixed, while being attached to the paper, in 3.7% paraformaldehyde for 1 h at room temperature, or, for γ-tubulin, in methanol for 20 min at −20°C. Resulting images were captured using N-SIM Nikon structured illumination microscope. TEM was performed as described (Dikranian et al, 2012).

### Recording of cilia beating and flow

Freshly dissected and flattened tracheas were incubated with solution of wheat germ agglutinin conjugated with Alexa 488 (1 μg/ml) in DMEM medium for 15 min at 37°C, a treatment that stained the cilia brightly fluorescent but did not alter the flow dynamics or the beat frequency. The specimens were then placed into a perfusion chamber with in- and out- tubing outlets which allowed the medium to flow through. To record the flow, fluorescent beads (0.2 μm FluoSpheres; Invitrogen) were added to the chamber. We used Bioptechs Focht Chamber System 3, a live-cell micro-observation chamber for upright microscopes. We used a 0.5-mm-thick 448910 gasket with 1-mm-wide slit or 0.25- or 0.5-mm-thick A2414 gaskets with 14-mm wide opening; this retained the trachea explants in place, while exposing them to the continuous flow of the pumped media. Externally pumped fluid flow is essential for supporting the basal level of consistent beating of the cilia for hours; otherwise, if the trachea explants are left in a closed chamber or without medium perfusion, the ciliary beat stops in several minutes (Winters et al, 2007). A miniature peristaltic pump (Model P720; Instech Laboratories) propelled high-glucose DMEM medium (without color pH indicator) at flow rates of 0.2 ml/h, a velocity that is an order magnitude lower than that of cilia-generated flow. This allowed for maintaining consistent ciliary besting for hours without interfering with the recording of the cilia-generated flow.

For drug treatments, a second identical pump was placed near the in-flow opening in the perfusion chamber. The pump was connected with the main flow tubing by the T-valve and was used for delivery of medium with the drug of choice. Switching the T-valve to the second pump outlet allowed the compound to enter the perfusion chamber quickly, with the focus of the microscope remaining on the same region or a group of cells. Such sequential recording of the same region before and after adding a compound of choice allowed for direct comparison of the changes in the same cells in response to treatment.

### Live high-speed fluorescence imaging

Live high-speed fluorescence imaging of tracheal cilia was accomplished with a system consisting of Axiophot microscope (Zeiss); X-Cite XYLIS LED light source (Excelitas Technologies), filter wheel 96A354-2 and MAC 6000 wheel controller (Ludl Electronic Products Ltd.), Prime sCMOS camera and DualView-lambda splitter (Photometrics), and optical filters and mirrors for Zeiss Fluorescence Cube Slider, splitter cube and filter wheel (CHROMA). The acquisition of movies was mediated by Micro-Manager 1.4 imaging software (Edelstein et al, 2014) and Dell Precision Tower 5810 computer. We typically acquired 16-bit 1,024 × 1,024 pixels fluorescence image sequences with 20× or 40× Plan-NEOFLUAR objectives and 10-ms exposure time at 65 fps recording for 5–10 s.

### Measurement of CBF

To determine the changes in CBF, we used quantification analysis tools available in ImageJ KymographBuilder, as applied to movie recordings made on several areas of each tracheal preparation. We routinely recorded about 10 movies on three tracheal explants for each genotype or treatment, quantified the average CBF after analyzing 30–50 cells in each preparation, and applied statistical analysis.

### Analysis of fluid flow dynamics

The .avi movie files of each experiment were imported into the Imaris software (Imaris 7.1.1; BitPlane Scientific Software). Bead movement was tracked using the “Track Spots Over Time” functionality of the software. 200 frames of video from each file were analyzed, correlating to 20 s of bead flow data. The number of tracks recorded per file ranged from 43 to 96. Imaris software calculated the average movement speed of each bead along its own track, as well as the overall displacement of each bead in the X- and Y-directions between the first frame in which it appeared, and the last. Beads speed data were exported into an Excel file (Excel 2010; Microsoft) and the speed and displacement of population of beads from each file were compared statistically. Bead displacement data were exported to MATLAB (MATLAB R2010b; Mathworks), and the angle of the displacement vector of each bead (relative to vertical) was calculated. To correct for minor differences in the orientation of the tissue samples between videos, the median displacement angle from each individual file was determined, and the distribution of individual displacement angles relative to the median was plotted as a histogram for each group. The distribution was fitted as Gaussian, and the deviation was calculated.

### Quantification of rotational polarity

To evaluate the changes in the basal bodies’ and cilia orientation, we used a plugin in the ImageJ suite, which allows for determining the angle between the longitudinal axis of the trachea and the vector connecting either the puncta of the Chibby and ODF2 signals in immunohistochemistry images or the basal body and basal foot in TEM micrographs. The collected data on the angles of basal body orientation in each cell relative to the longitudinal axis of the trachea were uploaded into the Oriana software suite for circular statistics and circular plots (KCS, Inc.), and this was followed by statistical analysis. In the presented circular plots, each vector represents one cell, the length of the vector indicates the polarity alignment within the cell, and the direction of the vector indicates the mean direction within each cell.

### Evaluation of the basal bodies-microtubules connections

We analyzed the microtubule network and the contacts between the microtubules and the basal feet using α-tubulin to mark microtubules and ODF2 to mark the basal feet. The changes in the microtubule network were examined by using sets of image stakes in the Z aspects. The contacts were categorized as (+) for the basal feet with a clear visible connection to the microtubules and as (−) for the basal feet not connected to the microtubules. The data from ∼50 cells of each genotype, with four to seven animals per group, were collected, averaged, statistically evaluated, and presented as graphs.

### Assessments of changes in the structure of the apical actin cortex

Structural changes in the cortical actin cytoskeleton and the regularity of the actin cortex architecture were compared and quantified by using the “Skeletonize (2D/3D)” plug-in for topological skeletonization in ImageJ (https://imagej.net/Skeletonize3D). We determined the statistical distribution of the length of the elementary actin branches forming the actin cortex (the “skeleton”) as they cross each other or are terminated. An organized structure is characterized by longer elements of the skeleton branches, whereas a more disordered structure of the actin cortex is characterized by shorter branches, which often terminate without forming a continuous and regular network. Quantification was based on 3D reconstructed images produced by N-SIM at ×100 magnification and collected for 20–50 cells from groups of five to seven animals in each cohort, that is, wild-type and nNOS mutants, before and after exposure to the NO donor SNAP and evaluated for statistical significance.

### Assessment of the Vangl1 distribution

For the quantitative analysis of the changes of Vangl1 distribution we determined the ratio of the Vangl1 signal in the membrane to that in the cytoplasm. We analyzed ∼50 cells per group (four to seven wild-type or mutant mice per group, with or without exposure to the NO donor) by using ImageJ and an in-house written plugin macro to quantify the Vangl1 signal associated either with the membrane (defined by a membrane marker Na/K ATPase) or the cytoplasm.

### Statistics and graphs

The statistical ANOVA test, *t* test, F-test, Wilcoxon test, and Pearson’s correlation test were performed in Excel, ImageJ, and Oriana. Graphs for the analysis were made in Excel and Adobe Illustrator. Graphs are presented as mean ± standard error of the mean. Statistically significant differences are indicated (*P < 0.05; **P < 0.01; ***P < 0.001).

## Supplementary Material

Reviewer comments

## References

[bib1] Avidor-Reiss T, Ha A, Basiri ML (2017) Transition zone migration: A mechanism for cytoplasmic ciliogenesis and postaxonemal centriole elongation. Cold Spring Harb Perspect Biol 9: a028142. 10.1101/cshperspect.a028142 28108487 PMC5538411

[bib2] Balligand JL, Feron O, Dessy C (2009) Enos activation by physical forces: From short-term regulation of contraction to chronic remodeling of cardiovascular tissues. Physiol Rev 89: 481–534. 10.1152/physrev.00042.2007 19342613

[bib3] Benink HA, Bement WM (2005) Concentric zones of active rhoa and cdc42 around single cell wounds. J Cell Biol 168: 429–439. 10.1083/jcb.200411109 15684032 PMC2171735

[bib4] Besson A, Gurian-West M, Schmidt A, Hall A, Roberts JM (2004) P27kip1 modulates cell migration through the regulation of rhoa activation. Genes Dev 18: 862–876. 10.1101/gad.1185504 15078817 PMC395846

[bib5] Boucher RC (2019) Muco-obstructive lung diseases. N Engl J Med 380: 1941–1953. 10.1056/NEJMra1813799 31091375

[bib6] Burke MC, Li FQ, Cyge B, Arashiro T, Brechbuhl HM, Chen X, Siller SS, Weiss MA, O’Connell CB, Love D, (2014) Chibby promotes ciliary vesicle formation and basal body docking during airway cell differentiation. J Cell Biol 207: 123–137. 10.1083/jcb.201406140 25313408 PMC4195830

[bib7] Bustamante-Marin XM, Ostrowski LE (2016) Cilia and mucociliary clearance. Cold Spring Harb Perspect Biol 9: a028241. 10.1101/cshperspect.a028241 PMC5378048 27864314

[bib8] Button B, Picher M, Boucher RC (2007) Differential effects of cyclic and constant stress on atp release and mucociliary transport by human airway epithelia. J Physiol 580: 577–592. 10.1113/jphysiol.2006.126086 17317749 PMC2075559

[bib9] Chen J, Zhang M (2013) The par3/par6/apkc complex and epithelial cell polarity. Exp Cell Res 319: 1357–1364. 10.1016/j.yexcr.2013.03.021 23535009

[bib10] Davis EE, Katsanis N (2012) The ciliopathies: A transitional model into systems biology of human genetic disease. Curr Opin Genet Dev 22: 290–303. 10.1016/j.gde.2012.04.006 22632799 PMC3509787

[bib11] Dikranian K, Kim J, Stewart FR, Levy MA, Holtzman DM (2012) Ultrastructural studies in app/ps1 mice expressing human apoe isoforms: Implications for alzheimer’s disease. Int J Clin Exp Pathol 5: 482–495. 22949930 PMC3430100

[bib12] Edelstein AD, Tsuchida MA, Amodaj N, Pinkard H, Vale RD, Stuurman N (2014) Advanced methods of microscope control using mumanager software. J Biol Methods 1: e10. 10.14440/jbm.2014.36 25606571 PMC4297649

[bib13] Fan S, Hurd TW, Liu CJ, Straight SW, Weimbs T, Hurd EA, Domino SE, Margolis B (2004) Polarity proteins control ciliogenesis via kinesin motor interactions. Curr Biol 14: 1451–1461. 10.1016/j.cub.2004.08.025 15324661

[bib14] Francis RJ, Chatterjee B, Loges NT, Zentgraf H, Omran H, Lo CW (2009) Initiation and maturation of cilia-generated flow in newborn and postnatal mouse airway. Am J Physiol Lung Cell Mol Physiol 296: L1067–L1075. 10.1152/ajplung.00001.2009 19346437 PMC2692794

[bib15] Gertsberg I, Hellman V, Fainshtein M, Weil S, Silberberg SD, Danilenko M, Priel Z (2004) Intracellular ca2+ regulates the phosphorylation and the dephosphorylation of ciliary proteins via the no pathway. J Gen Physiol 124: 527–540. 10.1085/jgp.200409153 15477378 PMC2234008

[bib16] Heiss C, Rodriguez-Mateos A, Kelm M (2015) Central role of enos in the maintenance of endothelial homeostasis. Antioxid Redox Signal 22: 1230–1242. 10.1089/ars.2014.6158 25330054 PMC4410282

[bib17] Herawati E, Taniguchi D, Kanoh H, Tateishi K, Ishihara S, Tsukita S (2016) Multiciliated cell basal bodies align in stereotypical patterns coordinated by the apical cytoskeleton. J Cell Biol 214: 571–586. 10.1083/jcb.201601023 27573463 PMC5004441

[bib18] Hildebrandt F, Benzing T, Katsanis N (2011) Ciliopathies. N Engl J Med 364: 1533–1543. 10.1056/NEJMra1010172 21506742 PMC3640822

[bib19] Hong Y (2018) Apkc: The kinase that phosphorylates cell polarity. F1000Res 7: F1000. 10.12688/f1000research.14427.1 PMC6020718 29983916

[bib20] Horani A, Ferkol TW, Dutcher SK, Brody SL (2016) Genetics and biology of primary ciliary dyskinesia. Paediatr Respir Rev 18: 18–24. 10.1016/j.prrv.2015.09.001 26476603 PMC4864047

[bib21] Hurt KJ, Sezen SF, Lagoda GF, Musicki B, Rameau GA, Snyder SH, Burnett AL (2012) Cyclic amp-dependent phosphorylation of neuronal nitric oxide synthase mediates penile erection. Proc Natl Acad Sci U S A 109: 16624–16629. 10.1073/pnas.1213790109 23012472 PMC3478647

[bib22] Hyndman KA, Bugaj V, Mironova E, Stockand JD, Pollock JS (2015) Nos1-dependent negative feedback regulation of the epithelial sodium channel in the collecting duct. Am J Physiol Renal Physiol 308: F244–F251. 10.1152/ajprenal.00596.2013 25391901 PMC4312960

[bib23] Jackson CL, Lucas JS, Walker WT, Owen H, Premadeva I, Lackie PM (2015) Neuronal nos localises to human airway cilia. Nitric Oxide 44: 3–7. 10.1016/j.niox.2014.11.003 25460324

[bib24] Jain B, Rubinstein I, Robbins RA, Leise KL, Sisson JH (1993) Modulation of airway epithelial cell ciliary beat frequency by nitric oxide. Biochem Biophys Res Commun 191: 83–88. 10.1006/bbrc.1993.1187 7680560

[bib25] Jiao J, Wang H, Lou W, Jin S, Fan E, Li Y, Han D, Zhang L (2011) Regulation of ciliary beat frequency by the nitric oxide signaling pathway in mouse nasal and tracheal epithelial cells. Exp Cell Res 317: 2548–2553. 10.1016/j.yexcr.2011.07.007 21787770

[bib26] Knowles MR, Zariwala M, Leigh M (2016) Primary ciliary dyskinesia. Clin Chest Med 37: 449–461. 10.1016/j.ccm.2016.04.008 27514592 PMC4988337

[bib27] Kunimoto K, Yamazaki Y, Nishida T, Shinohara K, Ishikawa H, Hasegawa T, Okanoue T, Hamada H, Noda T, Tamura A, (2012) Coordinated ciliary beating requires odf2-mediated polarization of basal bodies via basal feet. Cell 148: 189–200. 10.1016/j.cell.2011.10.052 22265411

[bib28] Li FQ, Chen X, Fisher C, Siller SS, Zelikman K, Kuriyama R, Takemaru KI (2016) Bar domain-containing fam92 proteins interact with chibby1 to facilitate ciliogenesis. Mol Cell Biol 36: 2668–2680. 10.1128/MCB.00160-16 27528616 PMC5064215

[bib29] Li FQ, Siller SS, Takemaru KI (2015) Basal body docking in airway ciliated cells. Oncotarget 6: 19944–19945. 10.18632/oncotarget.4609 26343519 PMC4652968

[bib30] Lobo J, Zariwala MA, Noone PG (2015) Primary ciliary dyskinesia. Semin Respir Crit Care Med 36: 169–179. 10.1055/s-0035-1546748 25826585 PMC4873960

[bib31] Lorenz R, Bertinetti D, Herberg FW (2017) Camp-dependent protein kinase and cgmp-dependent protein kinase as cyclic nucleotide effectors. Handb Exp Pharmacol 238: 105–122. 10.1007/164_2015_36 27885524

[bib32] Love D, Li FQ, Burke MC, Cyge B, Ohmitsu M, Cabello J, Larson JE, Brody SL, Cohen JC, Takemaru K (2010) Altered lung morphogenesis, epithelial cell differentiation and mechanics in mice deficient in the wnt/beta-catenin antagonist chibby. PLoS One 5: e13600. 10.1371/journal.pone.0013600 21049041 PMC2963606

[bib33] Melikian N, Seddon MD, Casadei B, Chowienczyk PJ, Shah AM (2009) Neuronal nitric oxide synthase and human vascular regulation. Trends Cardiovasc Med 19: 256–262. 10.1016/j.tcm.2010.02.007 20447567 PMC2984617

[bib34] Miller AL, Bement WM (2009) Regulation of cytokinesis by rho gtpase flux. Nat Cell Biol 11: 71–77. 10.1038/ncb1814 19060892 PMC2677303

[bib35] Mitchell B, Jacobs R, Li J, Chien S, Kintner C (2007) A positive feedback mechanism governs the polarity and motion of motile cilia. Nature 447: 97–101. 10.1038/nature05771 17450123

[bib68] Packer MA, Stasiv Y, Benraiss A, Chmielnicki E, Grinberg A, Westphal H, Goldman SA, Enikolopov G (2003) Nitric oxide negatively regulates mammalian adult neurogenesis. Proc Natl Acad Sci U S A 100: 9566–9571. 10.1073/pnas.1633579100 12886012 PMC170958

[bib37] Pan J, You Y, Huang T, Brody SL (2007) Rhoa-mediated apical actin enrichment is required for ciliogenesis and promoted by foxj1. J Cell Sci 120: 1868–1876. 10.1242/jcs.005306 17488776

[bib38] Park TJ, Mitchell BJ, Abitua PB, Kintner C, Wallingford JB (2008) Dishevelled controls apical docking and planar polarization of basal bodies in ciliated epithelial cells. Nat Genet 40: 871–879. 10.1038/ng.104 18552847 PMC2771675

[bib39] Peunova N, Scheinker V, Ravi K, Enikolopov G (2007) Nitric oxide coordinates cell proliferation and cell movements during early development of xenopus. Cell Cycle 6: 3132–3144. 10.4161/cc.6.24.5146 18073535

[bib40] Pruliere G, Cosson J, Chevalier S, Sardet C, Chenevert J (2011) Atypical protein kinase c controls sea urchin ciliogenesis. Mol Biol Cell 22: 2042–2053. 10.1091/mbc.E10-10-0844 21508313 PMC3113769

[bib41] Sato K, Yokota T, Ichioka S, Shibata M, Takeda S (2008) Vasodilation of intramuscular arterioles under shear stress in dystrophin-deficient skeletal muscle is impaired through decreased nnos expression. Acta Myol 27: 30–36. 19108575 PMC2859605

[bib42] Sedzinski J, Hannezo E, Tu F, Biro M, Wallingford JB (2017) Rhoa regulates actin network dynamics during apical surface emergence in multiciliated epithelial cells. J Cell Sci 130: 420–428. 10.1242/jcs.194704 28089989 PMC5278671

[bib43] Shu X, Keller TCS 4th, Begandt D, Butcher JT, Biwer L, Keller AS, Columbus L, Isakson BE (2015) Endothelial nitric oxide synthase in the microcirculation. Cell Mol Life Sci 72: 4561–4575. 10.1007/s00018-015-2021-0 26390975 PMC4628887

[bib44] Siller SS, Burke MC, Li FQ, Takemaru K (2015) Chibby functions to preserve normal ciliary morphology through the regulation of intraflagellar transport in airway ciliated cells. Cell Cycle 14: 3163–3172. 10.1080/15384101.2015.1080396 26266958 PMC4825556

[bib45] Sisson JH, Pavlik JA, Wyatt TA (2009) Alcohol stimulates ciliary motility of isolated airway axonemes through a nitric oxide, cyclase, and cyclic nucleotide-dependent kinase mechanism. Alcohol Clin Exp Res 33: 610–616. 10.1111/j.1530-0277.2008.00875.x 19183138 PMC2749507

[bib46] Taniguchi H, He M, Wu P, Kim S, Paik R, Sugino K, Kvitsiani D, Fu Y, Lu J, Lin Y, (2011) A resource of cre driver lines for genetic targeting of gabaergic neurons in cerebral cortex. Neuron 71: 995–1013. 10.1016/j.neuron.2011.07.026 21943598 PMC3779648

[bib47] Tarran R, Button B, Boucher RC (2006) Regulation of normal and cystic fibrosis airway surface liquid volume by phasic shear stress. Annu Rev Physiol 68: 543–561. 10.1146/annurev.physiol.68.072304.112754 16460283

[bib48] Veres TZ, Kopcsanyi T, Tirri M, Braun A, Miyasaka M, Germain RN, Jalkanen S, Salmi M (2017) Intubation-free in vivo imaging of the tracheal mucosa using two-photon microscopy. Sci Rep 7: 694. 10.1038/s41598-017-00769-6 28386104 PMC5429620

[bib49] Vladar EK, Antic D, Axelrod JD (2009) Planar cell polarity signaling: The developing cell’s compass. Cold Spring Harb Perspect Biol 1: a002964. 10.1101/cshperspect.a002964 20066108 PMC2773631

[bib50] Vladar EK, Bayly RD, Sangoram AM, Scott MP, Axelrod JD (2012) Microtubules enable the planar cell polarity of airway cilia. Curr Biol 22: 2203–2212. 10.1016/j.cub.2012.09.046 23122850 PMC3518597

[bib51] Vladar EK, Nayak JV, Milla CE, Axelrod JD (2016) Airway epithelial homeostasis and planar cell polarity signaling depend on multiciliated cell differentiation. JCI Insight 1: e88027. 10.1172/jci.insight.88027 27570836 PMC4996276

[bib52] Voronina VA, Takemaru K, Treuting P, Love D, Grubb BR, Hajjar AM, Adams A, Li FQ, Moon RT (2009) Inactivation of chibby affects function of motile airway cilia. J Cell Biol 185: 225–233. 10.1083/jcb.200809144 19364920 PMC2700371

[bib53] Walker WT, Jackson CL, Lackie PM, Hogg C, Lucas JS (2012) Nitric oxide in primary ciliary dyskinesia. Eur Respir J 40: 1024–1032. 10.1183/09031936.00176111 22408195

[bib54] Walker WT, Liew A, Harris A, Cole J, Lucas JS (2013) Upper and lower airway nitric oxide levels in primary ciliary dyskinesia, cystic fibrosis and asthma. Respir Med 107: 380–386. 10.1016/j.rmed.2012.11.021 23290188

[bib55] Wallingford JB (2006) Planar cell polarity, ciliogenesis and neural tube defects. Hum Mol Genet 15: R227–R234. 10.1093/hmg/ddl216 16987888

[bib56] Wallingford JB (2010) Planar cell polarity signaling, cilia and polarized ciliary beating. Curr Opin Cell Biol 22: 597–604. 10.1016/j.ceb.2010.07.011 20817501 PMC2974441

[bib57] Wallingford JB, Mitchell B (2011) Strange as it may seem: The many links between wnt signaling, planar cell polarity, and cilia. Genes Dev 25: 201–213. 10.1101/gad.2008011 21289065 PMC3034894

[bib58] Werner C, Onnebrink JG, Omran H (2015) Diagnosis and management of primary ciliary dyskinesia. Cilia 4: 2. 10.1186/s13630-014-0011-8 25610612 PMC4300728

[bib59] Werner ME, Hwang P, Huisman F, Taborek P, Yu CC, Mitchell BJ (2011) Actin and microtubules drive differential aspects of planar cell polarity in multiciliated cells. J Cell Biol 195: 19–26. 10.1083/jcb.201106110 21949415 PMC3187709

[bib60] Wheway G, Mitchison HM (2019) Opportunities and challenges for molecular understanding of ciliopathies-the 100,000 genomes project. Front Genet 10: 127. 10.3389/fgene.2019.00127 30915099 PMC6421331

[bib61] Winters SL, Davis CW, Boucher RC (2007) Mechanosensitivity of mouse tracheal ciliary beat frequency: Roles for ca2+, purinergic signaling, tonicity, and viscosity. Am J Physiol Lung Cell Mol Physiol 292: L614–L624. 10.1152/ajplung.00288.2005 16963528

[bib62] Wyatt TA (2015) Cyclic gmp and cilia motility. Cells 4: 315–330. 10.3390/cells4030315 26264028 PMC4588039

[bib63] Yoshimura S, Egerer J, Fuchs E, Haas AK, Barr FA (2007) Functional dissection of rab gtpases involved in primary cilium formation. J Cell Biol 178: 363–369. 10.1083/jcb.200703047 17646400 PMC2064854

[bib64] Zaoui K, Honore S, Isnardon D, Braguer D, Badache A (2008) Memo-rhoa-mdia1 signaling controls microtubules, the actin network, and adhesion site formation in migrating cells. J Cell Biol 183: 401–408. 10.1083/jcb.200805107 18955552 PMC2575782

[bib65] Zariwala MA, Knowles MR, Omran H (2007) Genetic defects in ciliary structure and function. Annu Rev Physiol 69: 423–450. 10.1146/annurev.physiol.69.040705.141301 17059358

[bib66] Zhang YH (2016) Neuronal nitric oxide synthase in hypertension: An update. Clin Hypertens 22: 20. 10.1186/s40885-016-0055-8 27822383 PMC5093926

