# Supplementary material for: nNOS regulates ciliated cell polarity, ciliary beat frequency, and directional flow in mouse trachea
Source: Life Sci Alliance. 2021 Mar 2;4(5):e202000981. doi: 10.26508/lsa.202000981 (PMC8008965; doi:10.26508/lsa.202000981)
Supplement: Reviewer comments [file LSA-2020-00981_review_history.pdf]

Life Science Alliance

nNOS REGULATES CILIATED CELL POLARITY, CILIARY BEAT FREQUENCY, AND DIRECTIONAL FLOW IN MOUSE TRACHEA

Anatoly Mikhailik, Tatyana Michurina, Krikor Takvor Dikranian, Stephen Hearn, Vladimir Maxakov, Saul Siller, Ken-Ichi Takemaru, Grigori Enikolopov and Natalia Peunova
DOI: https://doi.org/10.26508/lsa.202100981

Corresponding author(s): Natalia Peunova, Stony Brook University of NY

Review Timeline:

Submission Date:	2021-01-11
Editorial Decision:	2021-01-29
Revision Received:	2021-02-08
Accepted:	2021-02-09

Scientific Editor: Shachi Bhatt

Transaction Report:

(Note: With the exception of the correction of typographical or spelling errors that could be a source of ambiguity, letters and reports are not edited. The original formatting of letters and referee reports may not be reflected in this compilation.)

No full Peer Review Process File is available with this article, as the authors have chosen not to make the review process public in this case.

January 29, 2021

RE: Life Science Alliance Manuscript #LSA-2020-00981-T

Dr. Natalia Peunova
Stony Brook University of NY
Anesthesiology
Stony Brook
Stony Brook, NY 11794-5140

Dear Dr. Peunova,

Thank you for submitting your revised manuscript entitled "nNOS REGULATES CILIATED CELL POLARITY, CILIARY BEAT FREQUENCY, AND DIRECTIONAL FLOW IN MOUSE TRACHEA".

For a brief overview - this manuscript was reviewed by one of the Alliance journals, and revised accordingly. The authors transferred the revised manuscript to Life Science Alliance (LSA), along with the original reviewer comments and a point-by-point rebuttal to the reviewers' comments. LSA editors reached out to the original referees to review the manuscript.

As you will note from the reviewers' comments, the referees are mostly satisfied with how the manuscript has been improved post-revision. Reviewer 2 does have some concerns about the timing of the morphological rescue shown in Figure 8 and the sub-cellular localization of Vangl1 - both of which should be addressable with additional clarification, discussion or minor text changes. We would, thus, be happy to publish your paper in Life Science Alliance pending final revisions necessary to address these points raised by Reviewer 2 and meet our formatting guidelines.

Along with the points listed below, we also request you to attend to the following:

-please revise the legend for figure 4A, B; 5A, B; 9A so that all panels are introduced and in order

-In the manuscript text file, please place your main, supplementary figure legends after the references sections

-please add ORCID ID for the corresponding author-you should have received instructions on how to do so

-please add an Author Contributions in our system

-please add callouts for Figures 4Ba,b,c,d,e,f; 5Aa,b,c,d,e,f; 9Ca,b,c,d,e,f; 9Da,b; S1 A,B,C,D,E,F,G,H to your main manuscript text

-please use the [10 author names, et al.] format in your references (i.e. limit the author names to the first 10)

If you are planning a press release on your work, please inform us immediately to allow informing our production team and scheduling a release date.

To upload the final version of your manuscript, please log in to your account: https://lsa.msubmit.net/cgi-bin/main.plex

You will be guided to complete the submission of your revised manuscript and to fill in all necessary information. Please get in touch in case you do not know or remember your login name.

To avoid unnecessary delays in the acceptance and publication of your paper, please read the following information carefully.

A. FINAL FILES:

These items are required for acceptance.

-- An editable version of the final text (.DOC or .DOCX) is needed for copyediting (no PDFs).

-- High-resolution figure, supplementary figure and video files uploaded as individual files: See our detailed guidelines for preparing your production-ready images, https://www.life-science-alliance.org/authors

-- Summary blurb (enter in submission system): A short text summarizing in a single sentence the study (max. 200 characters including spaces). This text is used in conjunction with the titles of papers, hence should be informative and complementary to the title. It should describe the context and significance of the findings for a general readership; it should be written in the present tense and refer to the work in the third person. Author names should not be mentioned.

B. MANUSCRIPT ORGANIZATION AND FORMATTING:

Full guidelines are available on our Instructions for Authors page, https://www.life-science-alliance.org/authors

We encourage our authors to provide original source data, particularly uncropped/-processed electrophoretic blots and spreadsheets for the main figures of the manuscript. If you would like to add source data, we would welcome one PDF/Excel-file per figure for this information. These files will be linked online as supplementary "Source Data" files.

Submission of a paper that does not conform to Life Science Alliance guidelines will delay the acceptance of your manuscript.

It is Life Science Alliance policy that if requested, original data images must be made available to the editors. Failure to provide original images upon request will result in unavoidable delays in publication. Please ensure that you have access to all original data images prior to final submission.

The license to publish form must be signed before your manuscript can be sent to production. A link to the electronic license to publish form will be sent to the corresponding author only. Please take a moment to check your funder requirements.

Reviews, decision letters, and point-by-point responses associated with peer-review at Life Science Alliance will be published online, alongside the manuscript. If you do want to opt out of having the reviewer reports and your point-by-point responses displayed, please let us know immediately.

Thank you for your attention to these final processing requirements. Please revise and format the manuscript and upload materials within 7 days.

Thank you for this interesting contribution, we look forward to publishing your paper in Life Science Alliance.

Sincerely,

Shachi Bhatt, Ph.D.
Executive Editor
Life Science Alliance
https://www.lsajournal.org/
Tweet @SciBhatt @LSAjournal

February 9, 2021

RE: Life Science Alliance Manuscript #LSA-2020-00981-TR

Dr. Natalia Peunova
Stony Brook University of NY
Anesthesiology
Stony Brook
Stony Brook, NY 11794-5140

Dear Dr. Peunova,

Thank you for submitting your Research Article entitled "nNOS REGULATES CILIATED CELL POLARITY, CILIARY BEAT FREQUENCY, AND DIRECTIONAL FLOW IN MOUSE TRACHEA". It is a pleasure to let you know that your manuscript is now accepted for publication in Life Science. Congratulations on this interesting work.

The final published version of your manuscript will be deposited by us to PubMed Central upon online publication.

Your manuscript will now progress through copyediting and proofing. It is journal policy that authors provide original data upon request.

Reviews, decision letters, and point-by-point responses associated with peer-review at Life Science Alliance will be published online, alongside the manuscript. If you do want to opt out of having the reviewer reports and your point-by-point responses displayed, please let us know immediately.

IMPORTANT: If you will be unreachable at any time, please provide us with the email address of an alternate author. Failure to respond to routine queries may lead to unavoidable delays in publication.

Scheduling details will be available from our production department. You will receive proofs shortly before the publication date. Only essential corrections can be made at the proof stage so if there are any minor final changes you wish to make to the manuscript, please let the journal office know now.

DISTRIBUTION OF MATERIALS:

Authors are required to distribute freely any materials used in experiments published in Life Science Alliance. Authors are encouraged to deposit materials used in their studies to the appropriate repositories for distribution to researchers.

You can contact the journal office with any questions, contact@life-science-alliance.org

Sincerely,

Shachi Bhatt, Ph.D.
Executive Editor
Life Science Alliance
https://www.lsajournal.org/
Tweet @SciBhatt @LSAjournal
Interested in an editorial career? EMBO Solutions is hiring a Scientific Editor to join the international Life Science Alliance team. Find out more here - https://www.embo.org/documents/jobs/Vacancy_Notice_Scientific_editor_LSA.pdf
